# How the Design of Technology-Mediated Cognitive Training Paradigms Impacts Efficacy in Childhood Cancer Survivors: A Systematic Review

**DOI:** 10.3390/children13101397

**Published:** 2026-10-14

**Authors:** Rebekah J. O’Loughlin, Ines Semendric, Kenneth J. Pope, David A. Hobbs, Lyndsey E. Collins-Praino

**Affiliations:** 1 Medical Device Research Institute, College of Science and Engineering, Flinders University, 4 MAB Eastern Promenade, Tonsley, SA 5042, Australia; 2 School of Pharmacy and Biomedical Science, College of Health, Adelaide University, Adelaide, SA 5005, Australia; 3 Health Evidence Synthesis, Recommendations and Impact (HESRI), College of Health, School of Public Health, Adelaide University, Adelaide, SA 5005, Australia; 4 Medical Device Research Institute, College of Medicine and Public Health, Flinders University, Sturt Road, Bedford Park, SA 5042, Australia; 5 School of Allied Health and Human Performance, College of Health, Adelaide University, Adelaide, SA 5005, Australia

**Keywords:** cognitive training, paediatric, cancer, cancer-related cognitive impairment, chemobrain, cognitive rehabilitation

## Abstract

**Highlights:**

**What are the main findings?**

- Across the six included studies participants demonstrated varied cognitive improvement across cognitive domains, primarily in the domain of working memory, suggesting technology-mediated cognitive training has potential to improve cognition in this population.
- There is large variation in testing methods and trial design, combined with low certainty evidence, making it difficult to assess and identify the true value of this intervention for cognitive training in paediatric cancer survivors.

**What are the implications of the main findings?**

- Future studies should incorporate appropriate control groups, standardised comprehensive cognitive testing, multiple testing baselines and longitudinal testing.
- Cancer-related cognitive impairment can affect a broad range of cognitive domains, and the cognitive training programs should reflect this breadth.

**Abstract:**

**Background/Objectives**: Cancer and its subsequent treatments have been shown to cause significant cognitive impairment, known as Cancer-Related Cognitive Impairment (CRCI). CRCI impacts a range of cognitive domains, including working memory and executive function, attention, concentration, short-term memory, visuospatial control, problem solving, and information processing speed. Treatment options for these cancer sequelae are limited, with many patients never receiving any medical rehabilitation efforts. Current treatment recommendations include pharmaceutical intervention, cognitive training (CT), physical activity, and self-management strategies, with a prior meta-analysis in adults supporting CT as the most promising of these options. Thus, this systematic review aims to explore the current body of evidence for the use of CT as a treatment for childhood cancer survivors and assess the strengths and weaknesses of previously utilised programs to determine how to best test and optimise CT for this population. **Methods**: A systematic search was conducted using PubMed, Scopus, PsycINFO and CINAHL. The review was prepared following the Preferred Reporting Items for Systematic reviews and Meta-Analyses (PRISMA) 2020 reporting guidelines. **Results**: From an initial 6172 studies, six were included in the final review, covering 208 intervention participants who completed CT. CT platforms used by the included studies were all technology-mediated, and consisted of ‘Cogmed’ (*n* = 4), Attention Process Training (*n* = 1) and ‘Captain’s log’ (*n* = 1). Cognitive testing in the included studies covered three domains: working memory, attention and delayed recall (memory). Studies showed varied improvement across these domains, with most positive effects in the domain of working memory, although certainty of evidence across the included studies was all assessed as low or very low. **Conclusions**: This systematic review provides preliminary evidence for the use of CT in childhood cancer survivors, but also makes a range of recommendations for future studies in this population, given the current gaps in the literature. This includes utilisation of a control group, use of consistent cognitive testing protocols, and CT design that targets the diverse nature of the cognitive change seen in CRCI. Together, these recommendations will allow future studies to more fully probe the utility of CT as an intervention for this population, while also optimising its usage.

## 1. Introduction

Approximately 400,000 children aged 0–19 are diagnosed with cancer globally per year, with the most common types being leukaemia, brain tumour, and lymphoma [1]. Incidence rates of childhood cancer are highest in the first 5 years of life, when some of the most critical brain development is occurring [2,3]. The overall prognosis for childhood cancer has improved significantly in recent decades, with a 5-year survival rate greater than 83% in all childhood age ranges [4]. As a result of the improvement in survival rates, the importance of research into the long-term issues of survivorship is growing.

The long-term effects of paediatric cancer can impact multiple body systems, including the cardiovascular, central nervous, digestive, renal, endocrine, immune, musculoskeletal, reproductive, respiratory, and urinary systems [5]. Of these, one significant central nervous system (CNS) sequela is cognitive impairment, which is reported in 40–60% of childhood cancer survivors [6]. These impairments have been reported to impact a range of cognitive domains, including working memory and executive function, attention, concentration, short-term memory, visuospatial control, problem solving, and information processing speed [7,8,9,10]. Initially termed ‘chemobrain’ by survivors, this impairment is formally known as cancer-related cognitive impairment (CRCI). The colloquial term ‘chemobrain’ stems from the idea that the impairment was a side-effect of chemotherapy treatment. Addressing this idea, a study on cognitive deficits resulting from chemotherapy alone found that 67.3% of participants who were survivors of childhood acute lymphoblastic leukaemia were more than 1 standard deviation below the normative mean for sustained attention, with participants who received higher intensity therapy also being at greater risk for below-average outcomes in processing speed and academic abilities [11]. Nevertheless, it is now understood that CRCI does not result only from chemotherapy, but from one or more of several factors, including both the cancer itself and its treatment modalities: radiotherapy, chemotherapy and surgery [12,13,14]. For example, in the St Jude Lifetime Cohort Study of childhood acute lymphoblastic leukaemia survivors, cranial radiation therapy has been shown to impact intelligence, academic, and memory functions. The impact is dependent on age at time of diagnosis, being more pronounced in younger individuals and worsening with increased dose [15]. Detrimental effects can also be due to the cancer itself. In support of this, a study assessing cognitive impairment prior to treatment in children with cancer, with (*n* = 20) and without (*n* = 27) brain tumours, found that these individuals performed significantly worse than normative data in tests of intelligence, working memory, verbal memory, and attention, with CNS involvement further increasing impairment [16]. These detrimental effects may either be a result of direct effects from the cancer itself through inflammatory and neuroendocrine mechanisms [17], or indirect effects, such as coping with the diagnosis, or missing out on school [16,18].

The impacts of CRCI are significant, as survivors have reported a range of negative effects resulting from CRCI, including difficulties with information recollection, performing regular daily activities, mental stamina and managing workload [6]. These functional limitations can vary between people, but overall lead to challenges participating in daily life [6,19]. Childhood cancer survivors experience these functional challenges in ways that are unique, with schooling challenges, difficulties with social integration, and trouble completing fundamental daily tasks, such as self-hygiene tasks and handling of money [6,20,21,22,23], being commonly reported. This can have long-term consequences, with childhood cancer survivors reporting lower levels of educational attainment, lower levels of career success and higher levels of job discrimination [24,25].

Despite its impact, however, there are substantial limitations to current treatment options for paediatric CRCI. Pharmaceuticals, primarily repurposed CNS stimulants and drugs for dementia and memory impairments, have been studied as a potential solution [26], but the hesitancy of survivors to take more medications is a significant limitation to this treatment option. Conklin et al. [27] reported that approximately 35% of potential participants had their parents decline participation in a trial of a CNS stimulant medication for CRCI for this reason. Non-pharmaceutical treatment options include cognitive training (CT), physical activity and self-management strategies [26,28]. A network meta-analysis of neuropsychological interventions for adults with CRCI found that CT was the most effective intervention at improving objectively measured cognitive function, while individuals’ subjective perception of their cognition was most improved with cognitive behavioural therapy, followed by cognitive rehabilitation and CT [28]. CT is defined as repetitive practice in specific cognitive domains, aimed at improving that area of cognitive function [28,29]. Evidence has shown varied significant cognitive improvement across a variety of cognitive domains in a range of conditions, including Parkinson’s disease [30], attention-deficit/hyperactivity disorder [31,32] and Alzheimer’s disease [33], as a result of CT. Additionally, an online survey study found that 72% of adult participants who wanted support with their CRCI expected that this be in the form of CT [34]. However, the efficacy of CT for CRCI in childhood cancer survivors, first pioneered by Butler et al. [35], remains underexplored, with inconsistent results across studies and substantial heterogeneity between studies. Encouragingly, a 2016 review on CT programs for childhood cancer patients and survivors did report improvements in attention and working memory [36]. However, while CT programs completed at home, after treatment and with parent involvement appeared to be the optimal design, the authors concluded that data were too sparse at the time to meaningfully inform best practice guidelines. A subsequent review, published in 2026 [37], summarised the more recent literature, but did not provide any quantitative analysis. Additionally, they included studies with participants at all stages of cancer, studies specifically excluded in this review due to the confounding factors of worsening cognition at the same time as treatment. This current systematic review investigates these findings more deeply, by focusing on a more precise definition of cognitive training and systematically estimating standardised effect sizes. Specifically, we review the efficacy of CT programs as a treatment method for cognitive deficits in childhood cancer survivors and assess the strengths and limitations of particular programs through a discussion of the factors that may influence their outcomes, in order to formulate recommendations on how to best optimise CT research for this population.

## 2. Materials and Methods

This review has been conducted in accordance with an a priori protocol registered with PROSPERO (ID:CRD42023412052). This review has been prepared following the Preferred Reporting Items for Systematic Reviews and Meta-Analyses (PRISMA) 2020 reporting guidelines [38].

### 2.1. Inclusion Criteria

#### 2.1.1. Participants

Children ≤18 years old who have a previous cancer diagnosis of any type and who, at the time of the study, were in remission (no detectable disease) and had ceased cancer treatment were included in this review. There were no limitations on treatment or therapy type received, and all participants, regardless of evidence of cognitive impairment or time since treatment cessation or remission, were included. There were no restrictions on the geographical location of participants or the settings where they received their cognitive treatment.

#### 2.1.2. Types of Interventions

Included studies were required to implement a CT intervention. CT was defined as a repetitive training task that focused on specific cognitive domains, with the goal of objectively improving them. There was no limitation on the dose (i.e., number or frequency of sessions) of the intervention. Trials that investigated CT in conjunction with other interventions, where the effect of CT could not be isolated, were excluded to ensure the effect of the CT alone was being measured. Where studies also included other groups completing other interventions, data from these groups were not extracted.

#### 2.1.3. Types of Studies

Peer-reviewed randomised controlled trials (RCT), experimental and quasi-experimental studies that investigated the effectiveness of CT for childhood cancer survivors with full-text availability were included. Conference abstracts and reviews were excluded, although their reference lists were used for reference searching.

#### 2.1.4. Outcome of Interest

The included studies were required to include a cognitive outcome as assessed by an established neuropsychological measure, either of global cognition or within a specific cognitive domain (e.g., working memory, language, etc.). Studies that presented only self-reported measures of cognitive function were excluded.

#### 2.1.5. Search Strategy

**Figure 1:**
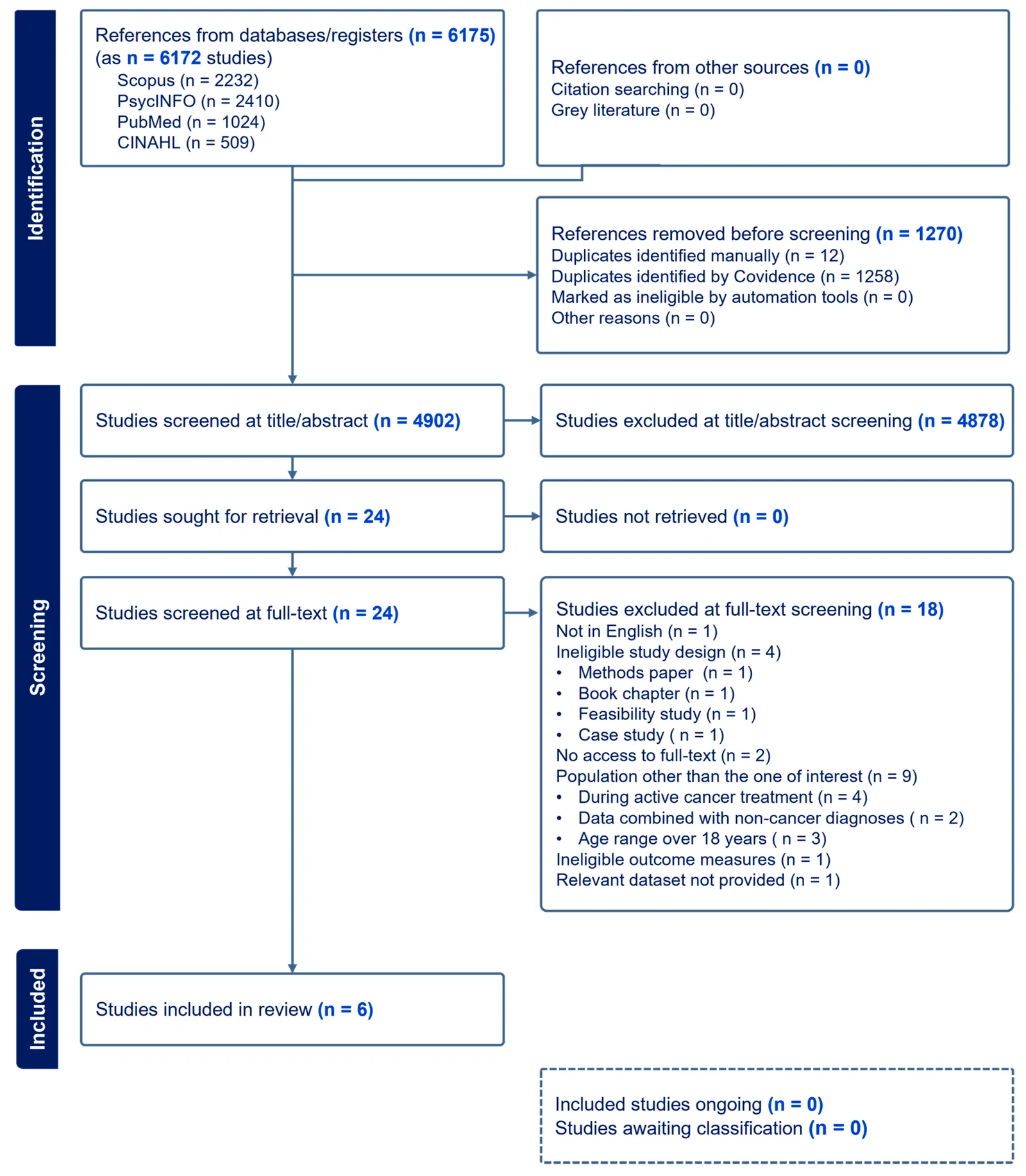
PRISMA diagram of study screening process.

Studies were sourced from four databases: PubMed, Scopus, PsycINFO and CINAHL (searches of all databases were completed October 2023 and renewed April 2026). The records returned for each search are outlined in Section 3, Figure 1.

PubMed was used to develop the initial search. The keywords and common language used in relevant articles identified through this search were subsequently used to identify primary search terms for a draft search strategy of all four databases. Adelaide University research librarian, Vikki Langton, assisted with creation and refinement of the search strategy (full search strategy, including the Boolean operators and field tags, specifically MeSH/subject headings, for all databases is in Supplementary Material S1). Search terms were broken up into three major categories: paediatric, cancer and cognitive impairment/remediation. Examples of synonyms/free-text words for each category include: child, adolescent, neoplasms, oncology, brain train, and cognitive remediation. The search terms, including all free-text words and keywords, were individually adapted for each database. The advanced search terms used as the basis for every database search are displayed in Table 1. No date limits were applied to these searches, and only English language publications were included. Reverse snowballing occurred through review of reference lists of all included studies.

#### 2.1.6. Study Selection

Following searches of the included databases, all articles were collated in Mendeley Reference Manager (www.mendeley.com, accessed on 12 October 2023). Articles were then imported into the Covidence systematic review tool (www.covidence.org, accessed on 12 October 2023), where duplicates were removed. Each study was screened by title and abstract, followed by whole text review. All reviews were judged against the inclusion and exclusion criteria by two independent reviewers (RO and IS) (Cohen’s Kappa: 0.71 title and abstract; 0.81 full-text). Reasons for exclusion in the full text review were recorded and included in the PRISMA diagram. In both screening stages, where conflicts arose, the two reviewers conferred to come to a consensus, and where consensus could not be met, a third reviewer (LCP) made the final decision. The results of all stages of the identification and removal process are reported in full in Section 3.

#### 2.1.7. Assessment of Methodological Quality

Two risk-of-bias tools were chosen for this review. The choice of two different tools was necessary as this review was not limited to RCTs. Where an included study was a RCT, the Cochrane Risk of Bias 2 (ROB 2) tool was used [39]. Where studies were non-randomised studies of interventions (NRSI), the Risk Of Bias In Non-randomised Studies-of Interventions (ROBINS-I) tool was used [40]. Assessment of methodological quality for all studies was completed by two independent reviewers (RO and IS). All assessments were completed before comparisons, and any conflicts were discussed between the two reviewers, and where consensus could not be met, a third reviewer (LCP) made the final decision. All studies, regardless of their methodological quality, underwent data extraction and synthesis where possible.

#### 2.1.8. Data Extraction

A data extraction template was created in Covidence (Supplementary Material S2), which was used for all data extraction. Two independent reviewers (RO and IS) completed all data extraction, and any conflicts were discussed between the two reviewers (89.6% proportionate agreement), and where consensus could not be met, a third reviewer (LCP) made the final decision. Extracted details included study design, study population, intervention design, timeline of data collection, and outcomes. All outcomes from every cognitive test performed in any study were extracted.

### 2.2. Data Synthesis

A narrative synthesis following SWiM guidelines [41] was conducted. Key study characteristics were tabulated to provide a detailed summary and results were grouped by cognitive domain, the most frequently used grouping method for cognitive results across the literature. The heterogeneity of the included studies, particularly with regard to the outcome measures, precluded the use of meta-analysis.

Standardised mean differences (Cohen’s *d*) and their 95% confidence intervals were used as the primary metrics for comparing findings across studies, in preference to relying on statistical significance alone. Where a study reported a standardised effect size directly, this was extracted as reported. Where raw group means, standard deviations and sample sizes were available, Cohen’s *d* was calculated as follows: for uncontrolled (single-arm) pre-post designs, *d* was calculated as the mean change from baseline to follow-up, divided by the pooled standard deviation of the baseline and follow-up scores; for controlled designs, *d* was calculated as the difference between the intervention and control groups’ change scores, divided by the sample-size-weighted pooled baseline standard deviation of the two groups.

Where a study reported an unstandardised effect estimate (e.g., an unstandardised regression coefficient), rather than group means and standard deviations suitable for calculating Cohen’s *d*, this estimate was extracted, along with its reported standard error and/or confidence interval, and reported separately. These were kept separate as they are not on a directly comparable scale to Cohen’s *d*, making direct comparison inappropriate.

95% confidence intervals for calculated *d* values were estimated using *d* and the relevant group sample sizes [42]. This approach assumes independence between time points and does not incorporate the correlation between repeated measures within participants; as pre-post correlations were not consistently reported across included studies, this is a conservative simplification consistent with common practice. Where 95% confidence intervals were reported within the publication, these were directly extracted, rather than being calculated. Where reported, *p* values were also extracted and are presented alongside effect estimates for descriptive purposes, but effect sizes were used to draw conclusions about intervention effectiveness.

### 2.3. Assessing Evidence Certainty

The Grading of Recommendations Assessment, Development and Evaluation (GRADE) tool was used for rating the certainty of evidence. Assessment methods were conducted following guidelines from Murad et al. [43] in rating the certainty of evidence in the absence of a single estimate of effect. Each outcome was assessed on the domains of risk of bias, consistency across studies, how direct the evidence answers the research question, and precision of the results. The final domain of GRADE, publication bias, was not assessed due to the small quantity of studies. A final ranking was given to each outcome to summarise these domains, reflecting the overall quality of evidence (very low, low, moderate and high). This process was piloted by one reviewer (RO) and assessed by a second reviewer (IS) and consensus between the reviewers was attained prior to completion. Where conflicts arose, they were resolved through discussion, and where consensus could not be met, a third reviewer (LCP) made the final decision. A Summary of Findings table was made through GRADEPro GDT software, (McMaster University, ON, Canada, 2026). The Summary of Findings table is detailed in Supplementary Material S5.

## 3. Results

### 3.1. Search Results

Searches identified 6172 studies (5591 October 2023; 581 April 2026), and removing 1270 (1182 October 2023; 88 April 2026) duplicates reduced the initial screening to 4902 studies overall (4409 October 2023; 493 April 2026). Title/abstract screening excluded a further 4878 studies, with 24 ultimately sought for retrieval. At full text screening, studies were excluded for not being in English, having an ineligible study design, lacking full-text access, including the wrong population, or not providing the relevant dataset after author contact was attempted. There were ultimately six studies, comprising nine unique publications (as four publications reported on the same sample population, with Conklin et al. [44] serving as the most representative publication on this work), which met the inclusion criteria and were included in this systematic review. The PRISMA diagram of included studies can be seen in Figure 1.

### 3.2. Quality Assessment

**Figure 2:**
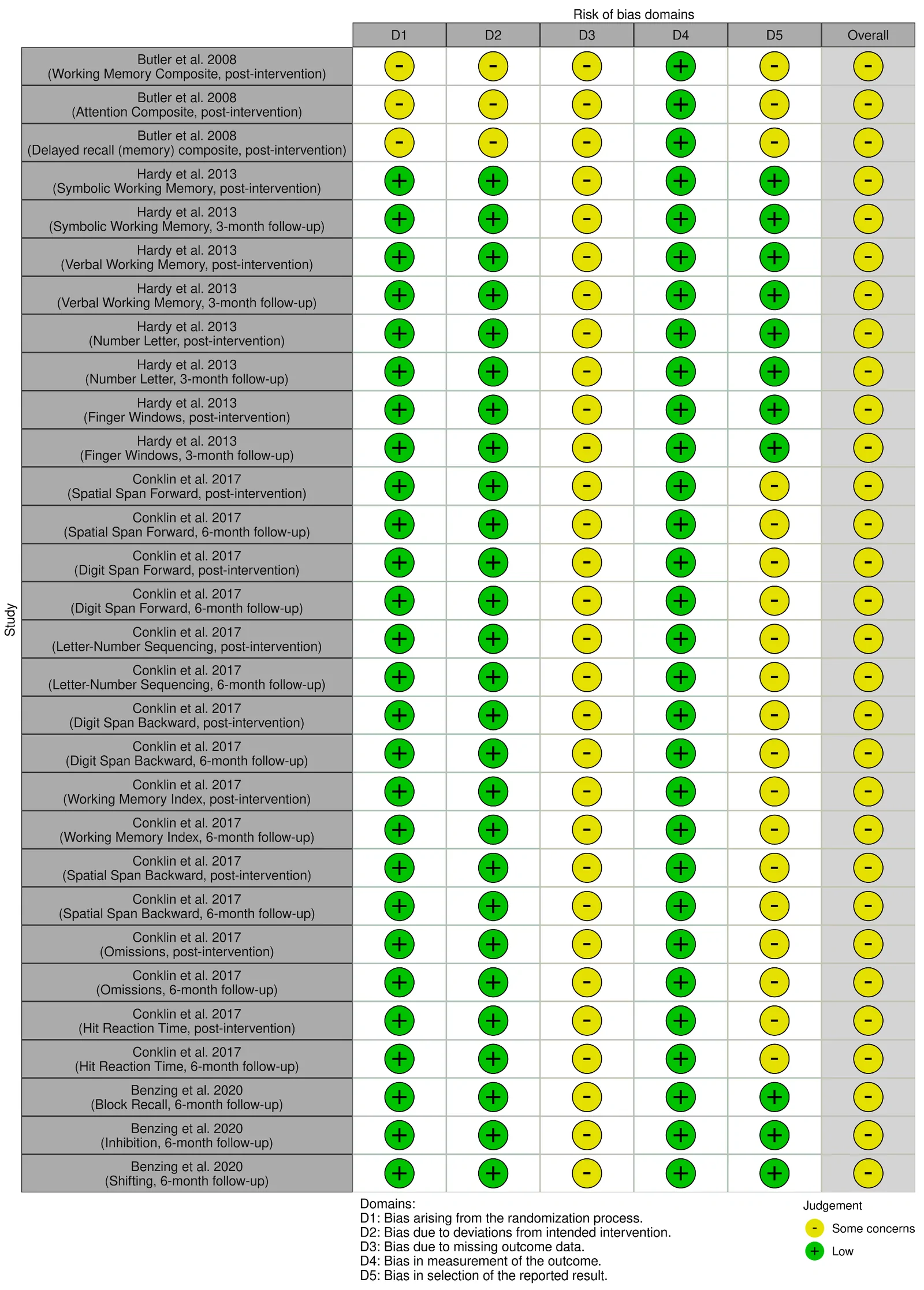
Summary of risk of bias of RCTs assessed using the Cochrane Risk of Bias 2.0 tool, at the outcome level [44,45,46,47].

**Figure 3:**
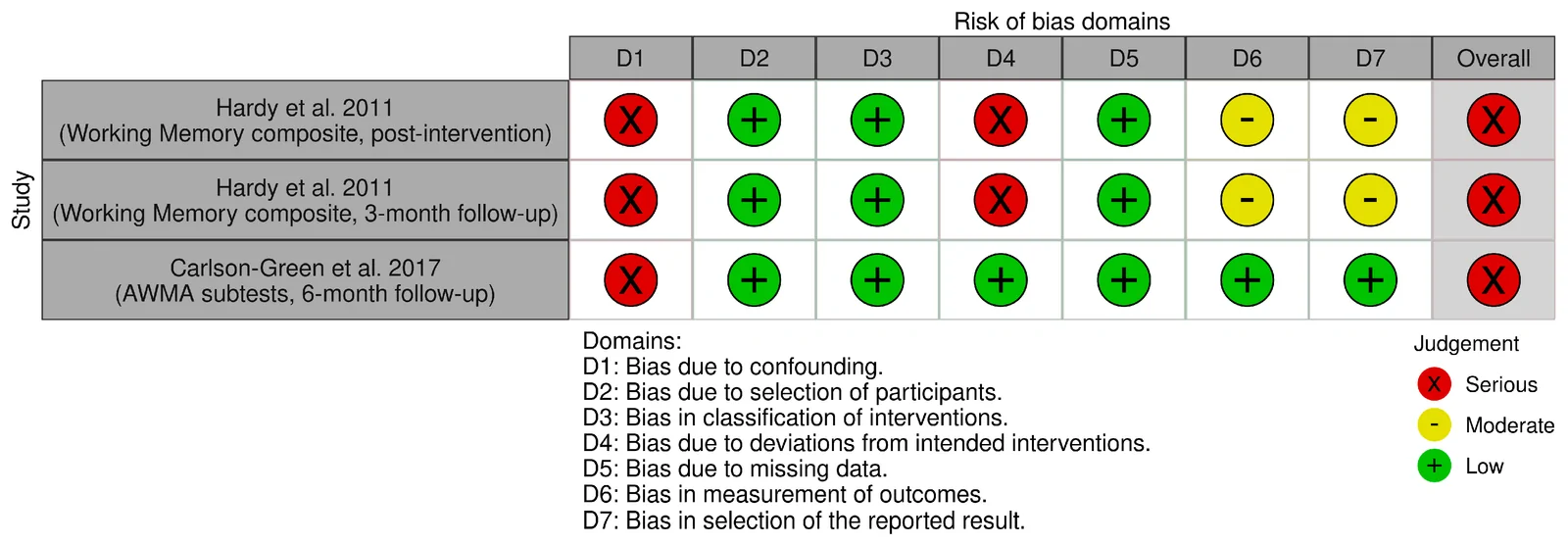
Summary of risk of bias of NRSIs assessed using the Risk of Bias In Non-randomised Studies-of Interventions, at the outcome level [48,49].

Risk of bias was assessed with the Cochrane ROB 2 tool [39] for randomised controlled trials and the ROBINS-I tool [40]. The results of these assessments are presented separately in Figure 2 and Figure 3, respectively. Full rationale for the individual domain judgements is provided in Supplementary Material S6.

All outcomes from the four RCTs were assessed as having some concerns regarding risk of bias (see Figure 2) [44,45,46,47]. Concerns primarily related to missing outcome data and selection of the reported result. Butler et al. [46] additionally raised concerns regarding the randomisation process and deviations from the intended interventions due to incomplete intervention adherence. Measurement of the cognitive outcomes was generally assessed at low risk of bias because outcomes were based on standardised, performance-based neuropsychological measures with objective scoring.

Outcomes from two NRSIs were assessed as having serious risk of bias [48,49] (see Figure 3). This was primarily due to confounding arising from the absence of a concurrent control group. Additional concerns related to deviations from intended interventions, measurement of outcomes, and selection of the reported result in Hardy et al. [48]. Carlson-Green et al. [49] was assessed at low risk of bias across the remaining domains.

### 3.3. Study and Participant Characteristics

The included studies comprised four RCTs and two self-controlled pilot trials, with publication dates ranging from 2008 to 2020. All studies, aside from one [47], were conducted in the United States, and all but one [46] were home-based.

The population sample included 208 intervention participants at baseline (42% female; *n* = 13 [45], *n* = 9 [48], *n* = 34 [44], *n* = 108 [46], *n* = 21 [49], and *n* = 23 [47]) and 118 control participants at baseline (39% female; *n* = 7 [45], *n* = 34 [44], *n* = 53 [46], and *n* = 24 [47]). Participants’ mean age at diagnosis ranged from 4.62 to 6 years (not reported in one study [48]), and ages at enrolment ranged from 7 to 18 years. Cancer diagnoses included acute lymphoblastic leukaemia (ALL) (*n* = 61), leukaemia and lymphoma more broadly (*n* = 27), medulloblastoma/primitive neuroectodermal tumour (*n* = 34), ependymoma (*n* = 13), glioma (*n* = 1), germinoma (*n* = 4), CNS tumours and neuroblastomas broadly (*n* = 9), as well as other cancers (*n* = 15), with one study not reporting cancer types [46]. Cancer treatments included radiation (cranial (*n* = 29), craniospinal (*n* = 8), conformal (*n* = 6), total body (*n* = 5), and unspecified location (*n* = 17)), chemotherapy (*n* = 122), surgery (*n* = 38), and bone marrow transplant (*n* = 5), with one study not reporting treatment population sizes [49] and another not reporting on treatments at all [46]. Time since treatment cessation in the intervention group ranged from 1–12 years, with a mean of approximately 5.24 years (6 ± 2.98 years [43], 5.7 ± 3.2 years (1–10 years) [48], 4.97 ± 3.02 years [44] and mean 5 years (1–12 years) [49]) with two studies not reporting this timeframe [46,47]. In the RCTs, time since treatment cessation in the control groups was 3 ± 1.77 years [45] and 5.04 ± 2.41 years [44], with this not reported in two studies [46,47]. For more detail on included participants see Supplementary Material S3.

### 3.4. Cognitive Training

Among the six included studies, all included CT systems were technology-mediated, also referred to as assistive technology for cognition, with three unique programs tested. Butler et al. [46] tested a custom Cognitive Remediation Program based around Attention Process Training [50], Hardy et al. [48] tested Captain’s Log (http://www.braintrain.com), a commercially available brain training program, and four studies implemented Cogmed (https://www.cogmed.com) [44,45,47,49], a clinically validated CT program [51]. The term technology-mediated is used to imply that technology is the means by which the training is conveyed to the user [52]. Details regarding the design features of these interventions were sourced from publicly available information. As all interventions are commercial products, comprehensive information about their underlying design was not publicly accessible.

The CT therapy dosage that each study implemented ranged from 5–8 weeks to 4–5 months, with weekly training sessions ranging from 50 min to four 2 h sessions. Compliance was defined differently in each study; this review will report compliance based on the study-specific pre-defined compliance. Compliance ranged from 44% to 90.5%. For more detail on these studies, see Table 2 and Supplementary Material S3.

Attention Process Training, implemented by Butler et al. [46], is a training program for mild to severe brain injury. It is delivered with a combination of digital and paper-based activities and is entirely self-contained. It was designed for the retraining of attention and concentration deficits. It is based around visual and auditory tasks that address sustained, selective, alternating and divided attention [50]. The difficulty of the tasks is modified throughout the training, based on the user’s abilities. Participants were instructed to use the system once per week for 20 sessions, where each session lasted 2 h and was completed in an outpatient clinical setting.

Captain’s Log [53], implemented by Hardy et al. [48], is a computer-based gamified CT system designed to target a range of cognitive domains, including processing speed, working memory, attention, and concentration. Difficulty is adjusted within the program based on user progress, increasing the customisation and personalisation of the system. The program also includes optional video games, provided to offer breaks in the training program. Participants were instructed to use the system for at least 50 min per week, for 12 weeks, at home.

The final CT tool implemented within this review is Cogmed [44,45,47,49]. Cogmed is a commercial working memory and attention training system delivered on any web-based device [51]. It involves game-like repetitive activities that target specific cognitive domains, with difficulty levels adjusted to the user’s needs. Carlson-Green et al. [49] instructed participants to use the system for 35 sessions over 8–12 weeks, while Conklin et al. [44] instructed participants to use the system for 30–45 min each time, in 25 sessions over 5–9 weeks, Hardy et al. [45] tasked participants to use the system for 25 sessions over 5–8 weeks, and Benzing et al. [47] had participants train three times per week, for 45 min each time across 8 weeks. These studies all instructed participants to choose when to engage with the program in their own home.

**Table 2:** Cognitive Training summaries.

Study	Cognitive Domains Targeted	Cognitive Domains Assessed	Cognitive Assessments
Butler et al. [46]	Focused, sustained, selective, alternating, and divided attention	Working memory, attention, immediate/delayed recall	Conners’ Continuous Performance Test-II—omissions, hit reaction time, variability RAVLT ^1^—trial 1, delay, trial 5 WRAML ^2^—sentence memory Children’s Memory Scale—stories immediate, stories delayed Rey-Osterrieth Complex Figure—Delay Stroop Color-Word Test—Trial 3 Trail Making Test B Brief Test of Attention—sum WISC III ^3^—digit forward, digit backward
Hardy et al. [48]	Attention, working memory, concentration, memory, basic problem-solving/reasoning skills, processing speed	Working memory	WISC-IV ^3^ digit span forward, digit span backward, letter-number sequencing, working memory index
Hardy et al. [45]	Visuo-spatial, verbal, and auditory working memory	Working memory	WRAML2 ^2^—symbolic working memory, verbal working memory, number letter, finger windows
Conklin et al. [44,54], Fournier-Goodnight et al. [55], Mendoza et al. [56]	Visuo-spatial, verbal and auditory working memory	Working memory, attention	WISC-IV ^3^—integrated spatial span forward, integrated spatial span backward, digit span forward, digit span backward, letter number sequencing, working memory index Conners’ Continuous Performance Test-II—omissions and hit reaction time
Carlson-Green et al. [49]	Visuo-spatial, verbal and auditory working memory	Working memory	AWMA ^4^—digit recall, word recall, counting recall, backwards digit recall, dot matrix, block recall, Mr. X, spatial recall
Benzing et al. [47]	Visuo-spatial, verbal and auditory working memory	Working memory	WMTB-C ^5^—block recall test D-KEFS ^6^—colour word interference test (inhibition and shifting)

^1^ RAVLT—Rey Auditory Verbal Learning Test; ^2^ WRAML—Wide Range Assessment of Memory and Learning; ^3^ WISC—Wechsler Intelligence Scale for Children; ^4^ AWMA—Automated Working Memory Assessment; ^5^ WMTB-C—Working Memory Test Battery for Children; ^6^ D-KEFS—Delis–Kaplin Executive Function System.

### 3.5. Intervention and Cognitive Outcome Results

Cognitive results are broken down into the cognitive domains tested in the included studies: working memory/executive function, attention, and delayed recall (memory). Supplementary Material S4 contains detailed plots of the effect estimates.

Included studies all used different statistical models and reported outcomes in different formats, although *p* values were reported by all. Effect estimates and confidence intervals were not consistently reported and were therefore calculated from available data for synthesis across studies, as described in Section 2.2. Consequently, results of this review show some significant *p* values, while confidence intervals for the same test include 0. This has occurred where *p* values were derived from the statistical models used in the included studies (e.g., linear mixed models [48], general linear models [45], and linear mixed effect models [44,49]), while confidence intervals were calculated for this review from published raw data or effect estimates. As these values were derived using different methods, complete agreement between *p* values and confidence intervals was not always expected.

#### 3.5.1. Working Memory and Executive Function

Working memory, often grouped with executive function, was the most commonly assessed cognitive domain, with all six studies including measures of working memory/executive function. Results across studies are summarised in Table 3. Cognitive training shows the most consistent evidence of benefit in this domain (and this is the domain best assessed), but the effect is not uniform. Effects cluster mainly on span-based working memory tasks, particularly spatial span, with 12 of 25 estimates having a 95% CI that excludes 0 (i.e., reaching statistical precision), with all favouring training. The signal is strongest for three of the Cogmed-based programs [44,47,49], moderate and inconsistent for Captain’s Log [48] and the Hardy et al. Cogmed study [45], and absent in the one large-sample composite WM measure [46].

Butler et al. [46] saw a significant improvement with a small effect in working memory (see Table 3 for specific subtests) within participants immediately following completion of the intervention (*p* < 0.001, *d* = 0.28, 95% CI [0.14, 0.42]), but this change was not maintained when compared to wait-listed controls (*p* = 0.194, *d* = 0.15, 95% CI [−0.07, 0.37]). This suggests that similar changes in cognition occurred in the control group of this study. Possible reasons are potential organic improvement in cognitive function over time, learning effects with the cognitive testing protocol used, or a placebo effect [57]. It was unfortunately not possible to draw conclusions about benefits beyond the immediate post-intervention period, as no long-term assessments were included in the analysis, given that the control group did not complete this measure.

Hardy et al. [48] also saw significant improvements with moderate effects in working memory (see Table 3 for specific subtests) within participants immediately following completion of the intervention, but this study did not include a control group, meaning that potential practice and placebo effects cannot be assessed. The persistence of this improvement was tested at 3-month follow-up and strengthened in the digit span task (*d* = 0.89 95% CI [0.05, 1.72]). In line with this Carlson-Green et al. [49] tested long-term improvement in a 6-month follow-up using the Automated Working Memory Assessment and found significant improvements within participants in six of eight subtests (see Table 3 for subtest breakdown); however, this result was also not compared to a control group and, as such, the impact of any potential learning or placebo effects cannot be determined. It is important to highlight the lack of control groups in these studies, meaning efficacy cannot be confirmed.

In contrast to these other studies, Hardy et al. [45] and Conklin et al. [44] tested both immediate and long-term improvement in working memory when compared to a control group. Conklin et al. [44] had a waitlist control group of cancer survivors, while Hardy et al. [45] included an active control group of cancer survivors who completed the same intervention, but without a difficulty progression. Hardy et al. [45] saw a significant improvement in the Wide Range Assessment of Memory and Learning (Second Edition) (WRAML2) Symbolic Working Memory test immediately post-intervention in the linear model when compared to the active control group, although this difference was not maintained at three-month follow-up. Despite this, effect size was large at both time points, in fact it strengthened at the long-term follow-up (*d* = 0.94 95% CI [−0.11, 1.99] post intervention; *d* = 1.22 95% CI [0.13, 2.31] long-term). Further, no difference was seen either immediately post-intervention or at three-month follow-up on the Verbal Working Memory, Number-Letter, or Finger Windows of the WRAML2, further emphasising that the effects may be subtest specific. In line with this, Conklin et al. [44] also saw a significant improvement and moderate effect sizes with 95% confidence intervals that exclude 0 immediately post-intervention in three of their working memory subtests (Spatial Span Backward *d* = 0.77 95% CI [0.25, 1.29], *p* = 0.001; Spatial Span Forward *d* = 0.71 95% CI [0.20, 1.23], *p* = 0.014; and Digit Span Backward *d* = 0.54 95% CI [0.03, 1.05], *p* = 0.043; of Wechsler Intelligence Scale for Children, Version 4 (WISC-IV)), but not on the Letter-Number Sequencing Task and Digit Span Forward of the WISC-IV. Durability of the long-term effects across the different studies is mixed, with some gains persisting long-term (Conklin et al. [44] Spatial Span Backward; *d* = 0.72 95% CI [0.21, 1.24]), while others that were significant post-intervention fade by follow-up (e.g., Conklin et al. [44] Spatial Span Forward, Digit Span Backward).

Executive function is essentially unassessed, with only one measure (Benzing et al. [47] Delis–Kaplan Executive Function System (D-KEFS) CWIT), reaching the calculated-effect-size stage, and it shows no effect, so no domain-level conclusion can be drawn for executive function specifically. These measures had estimates that were of identical magnitude and opposite directions. As these outcomes were derived from related D-KEFS tasks, the opposing signs may result from different outcome coding or scoring direction; however, the available data was not sufficient to provide a conclusion.

Overall, results across studies varied substantially, but seem to indicate that working memory improvement is possible with CT in this population, although additional studies with active control groups and long-term follow-up time points, which include assessment of transfer effects to other cognitive domains and global cognition, are essential to fully assess these potential benefits.

**Table 3:** Working memory and executive function result breakdown.

Study	Outcome	Post *d/β* (95% CI)	Post *p*	LTFO ^3^ *d/β* (95% CI)	LTFO *p* ^3^
Butler et al. [46] ^1^	Working memory (composite)	0.15 (−0.07, 0.37)	0.194	—	
Hardy et al. [48] ^2^	WISC-IV ^4^ WM ^5^ index	0.63 (−0.10, 1.35)	**0.03**	0.65 (−0.12, 1.42)	0.07
	WISC-IV ^4^ Digit Span Forward	0.73 (−0.02, 1.47)	**0.01**	**0.89 (0.05, 1.72)**	**<0.01**
	WISC-IV ^4^ Digit Span Backward	0.27 (−0.39, 0.94)	>0.05	0.41 (−0.32, 1.13)	>0.05
	WISC-IV ^4^ Letter-Number Seq.	0.23 (−0.43, 0.89)	>0.05	0.34 (−0.38, 1.05)	>0.05
Hardy et al. [45] ^2^	WRAML-2 ^6^ Symbolic WM	0.94 (−0.11, 1.99)	**0.05**	**1.22 (0.13, 2.31)**	0.08
	WRAML-2 ^6^ Verbal WM ^2^	−0.12 (−1.12, 0.88)	0.71	−0.21 (−1.21, 0.79)	0.65
	WRAML-2 ^6^ Number-Letter	0.28 (−0.72, 1.28)	0.58	−0.05 (−1.04, 0.94)	0.90
	WRAML-2 ^6^ Finger Windows	0.67 (−0.35, 1.69)	0.18	0.30 (−0.70, 1.30)	0.59
Conklin et al. [44,54], Fournier-Goodnight et al. [55], Mendoza et al. [56] ^1^	WISC-IV ^4^ Spatial span forward	**0.71 (0.20, 1.23)**	**0.014**	0.14 (−0.36, 0.63)	**0.049**
	WISC-IV ^4^ Digit span forward	0.03 (−0.47, 0.53)	0.897	0.12 (−0.38, 0.62)	0.561
	WISC-IV ^4^ Letter-number seq.	0.32 (−0.18, 0.82)	0.088	0.33 (−0.17, 0.83)	0.948
	WISC-IV ^4^ Digit span backward	**0.54 (0.03, 1.05)**	**0.043**	0.26 (−0.24, 0.76)	0.315
	WISC-IV ^4^ WM ^5^ index	0.38 (−0.12, 0.89)	**0.017**	0.34 (−0.16, 0.84)	0.923
	WISC-IV ^4^ Spatial span backward	**0.77 (0.25, 1.29)**	**0.001**	**0.72 (0.21, 1.24)**	0.940
Carlson-Green et al. [49] ^1^	AWMA ^7^ Digit Recall	—	—	**8.7 (2.82, 14.58)**	**0.009**
	AWMA ^7^ Word Recall	—	—	**9.3 (1.26, 17.34)**	**0.035**
	AWMA ^7^ Counting Recall	—	—	−2.1 (−7.39, 3.19)	0.457
	AWMA ^7^ Backwards Digit	—	—	**7.8 (0.35, 15.25)**	**0.052**
	AWMA ^7^ Dot Matrix	—	—	**16.8 (7.98, 25.62)**	**0.002**
	AWMA ^7^ Block Recall	—	—	**8.7 (3.41, 13.99)**	**0.005**
	AWMA ^7^ Mr. X	—	—	**7.0 (2.1, 11.9)**	**0.013**
	AWMA ^7^ Spatial Recall	—	—	**12.3 (3.87, 20.73)**	**0.010**
Benzing et al. [47] ^2^	WMTB-C ^8^ Block Recall	—	—	**3.3 (0.74, 5.85)**	**0.012**
	D-KEFS CWIT ^9^ Inhibition	—	—	0.23 (−0.12, 0.58)	0.193
	D-KEFS CWIT ^9^ Shifting	—	—	−0.23 (−0.59, 0.12)	0.200

^1^ Follow-up at 6-months post-intervention, ^2^ Follow-up at 3-months post-intervention, ^3^ LTFO—Long-Term Follow-up; ^4^ WISC-IV—Wechsler Intelligence Scale for Children, 4th Edition; ^5^ WM—Working Memory; ^6^ WRAML-2—Wide Range Assessment of Memory and Learning, 2nd edition; ^7^ AWMA—Automated Working Memory Assessment; ^8^ WMTB-C—Working Memory Test Battery for Children; ^9^ D-KEFS CWIT—Delis–Kaplin Executive Function System Color Word Interference Test. Bold values indicate statistically significant results.

#### 3.5.2. Attention

Two studies included a measure of attention in their cognitive testing, with one reporting moderate effects with significant improvements in attention at one time point when compared to controls. Results across studies are summarised in Table 4. Butler et al. [46] reported a significant improvement with small effects in focused attention in the intervention group immediately post-intervention when compared to their pre-intervention baseline (*p* < 0.001, *d* = 0.31, 95% CI [0.18, 0.44]). When compared to the waitlist control group, however, this effect was not maintained (*p* = 0.879, *d* = 0.02, 95% CI [−0.19, 0.23]). Conklin et al. [44] was the only study that reported a significant improvement immediately post-intervention across two different measures of attention—Omissions and Hit Reaction Time on Conners’ Continuous Performance Test (CPT-II)—when compared to their waitlist control group. Only one of these estimates reached statistical precision (CPT-II Omissions *d* = −0.61 95% CI [−1.12, −0.10]) but this is not replicated on the companion Hit Reaction Time measure and neither persists to long-term follow-up. This result is also not corroborated by Butler at al. [48], a study with a much larger sample size. Taken together, the domain of attention appears to have a null-to-negligible effect in these studies, with the minimal and inconsistent findings most plausibly a fluctuation rather than a robust signal. Nevertheless, improvement in attention cannot be ruled out given how few attention-specific outcomes were captured overall.

#### 3.5.3. Delayed Recall (Memory)

Only one study assessed changes in delayed recall (i.e., memory) following engagement with CT. Butler et al. [46] detected a negligible effect estimate (*d* = −0.11 95% CI [−0.36, 0.14]) when comparing the intervention group with controls post-intervention. Results are summarised in Table 5. This review does not show any evidence of benefit, but this result rests on a single study, with a single composite estimate. This limitation means that no conclusion can be made about the effect of CT in this population and more well-powered studies are essential.

#### 3.5.4. Overall Efficacy

Cognitive training in paediatric cancer survivors appears to show domain-specific rather than general benefit. The strongest finding across studies was an improvement in working-memory span tasks (especially spatial span), with no consistent benefit for attention and insufficient data to judge executive function or memory. Even for working memory, it must be acknowledged that the effects are frequently imprecise, with wide confidence intervals derived from small samples, that often do not persist to long-term follow-up. Given this, while cautiously promising, at the current time, the literature is too sparse and has too many caveats to definitively draw a conclusion about the benefit of cognitive training for improving cognitive function overall in paediatric cancer survivors.

### 3.6. GRADE Certainty Assessment

Outcomes from the included studies were divided into three cognitive domains: (1) executive function and working memory; (2) attention; and (3) delayed recall (memory). These domains were then further divided into the immediate follow-up and long-term follow-up time points and grouped into RCTs and non-randomised studies of interventions. Concerns predominantly arose from the risk of bias and data imprecision from confidence intervals that included a range of effects, from potential benefit to potential harm. These results indicate a low level of certainty in the RCTs that assessed working memory and executive function at both time points, as well as the immediate follow-up of attention and delayed recall (memory). Very low level of certainty was determined in both of the non-randomised groups (working memory and executive function at both time points) as well as the long-term follow-up for attention in RCTs. These findings suggest that substantial caution should be taken when interpreting these results. Additionally, all domain assessments were made on three or fewer studies, with three groupings only including a single study, further emphasising the need to interpret findings with caution. For more information detailing the full justification of these assessments see Supplementary Material S5.

## 4. Discussion

The aim of this review was to assess the efficacy of CT programs as a remediation method for childhood cancer survivors and to assess the strengths and limitations of specific studies. CT has previously been used to target cognitive impairment in a variety of neurological disorders that involve cognitive dysfunction [28,30,31,32,33,36]. In general, it has been reported to have a positive impact on cognition [29,36,57,58,59,60,61], but there is limited prior research on how best to optimise its implementation and design for CRCI, particularly in the paediatric population. This discussion aims to break down the different research methodologies and CT design features, where publicly available, that may impact the perceived and real efficacy in this population.

When considering this population, it is important to note that evidence suggests that both cancer type and specific treatment received can influence the severity of cognitive impairment experienced by survivors of childhood cancer [62]. The most common childhood cancer is ALL, followed by CNS cancers [3,63], and this was reflected in the studies included in this review, with all but one limiting participants to brain tumour and/or ALL survivors. Notably, survivors of CNS tumours are inherently at greatest risk of cognitive impairment, given that tumour location, brain surgery and/or cranial radiation are all found to be risk factors for worse neurocognitive outcomes in childhood cancer survivors [16,63,64]. Nevertheless, CNS-directed treatments, such as cranial radiation and intrathecal chemotherapy, are also standard procedure in non-CNS cancers, to minimise risk of future CNS relapse, and a recent review from our group has shown that a significant percentage of survivors of paediatric non-CNS cancers still experience cognitive impairment across a range of domains [65]. Given this, it is important to consider whether individuals with a history of specific types of cancer or treatment protocols may benefit preferentially from CT. Unfortunately, the studies included in this review did not report results on specific diagnoses/treatment types, precluding such analyses in the current work.

Despite this limitation, we were still able to assess the potential benefits of CT for childhood cancer survivors more broadly. Small, moderate and large effects were detected in multiple studies, primarily focused on working memory improvements (although it must be acknowledged that there was serious [48,49] risk of bias in two out of the six included studies and some concerns in the other four [44,45,46,47] and there were concerns about the certainty of the evidence more generally, as evidenced by the GRADE assessment, meaning that these improvements must be interpreted with caution). These improvements were found across two of the cognitive domains that were measured: working memory/executive function and attention, although the findings in the attention domain were not consistent across studies, and likely not a true effect. CRCI can impact a range of cognitive domains, with deficits in executive function, working memory, attention, visuospatial/perceptual motor function, language and psychomotor processing speed all previously reported in childhood cancer survivors [16,64,65,66,67]. Similarly, the studies included in the current review trained a diverse subset of cognitive domains, with one study training focused, sustained, selective, alternating, and divided attention [46], one processing speed, working memory, visuospatial skills, attention and concentration [48], and the final four working memory and attention [44,45,47,49]. Notably, while both working memory and attention/concentration were domains commonly targeted across studies, some aspects of cognitive function reported to be affected by CRCI in childhood cancer survivors were less commonly addressed, with only the study by Hardy et al. [48] including processing speed as targeted domains for their CT. Other domains, such as language and long-term memory, appear to have been omitted from training entirely. Thus, the CT paradigms included within the current review may not address the full range of cognitive changes potentially seen in childhood cancer survivors. Further, the variability in domains addressed by the CT paradigms included in the current review makes it difficult to directly compare benefits observed between studies. To address this, it is recommended that future CT systems intended for use with this population be specifically designed to consider the breadth of cognitive domains potentially impacted by CRCI.

When assessing the potential benefits of a given CT intervention, it is also important to ensure that the cognitive assessments selected are appropriate for investigating the specific domains targeted by the intervention. This was not always the case with the studies included in the current review. For example, although delayed recall (i.e., memory) was assessed within one study [46], it was not included as a specific training domain within that study’s CT program, or any other included CT program. Similarly, although Hardy and colleagues included processing speed as a targeted domain within their CT system, they did not specifically assess for improvements in these areas [48]. To improve this alignment, it may be beneficial to draw upon the recommendations of the International Cognition and Cancer Task Force (ICCTF) for testing for the cognitive sequelae of cancer and its treatment in adults with non-CNS tumours [68]. While these recommendations may not be directly applicable to childhood cancer survivors, they still offer critical guidance that may be adaptable for this population.

More specifically, the ICCTF recommends that tests, at a minimum, target the cognitive domains of learning and memory, processing speed and executive function, as these are the cognitive domains most affected in CRCI, including in childhood cancer survivors [16,64,65,66,67]. Assessments must also have strong test-retest reliability, be suitable for multinational application and be available in alternate forms, to reduce the risk of practice effects over time. Finding tests that meet these criteria and that are specifically validated for use in a paediatric population is therefore critical both for standardising the investigation of the cognitive sequelae in paediatric CRCI and for assessing the benefits of potential treatment modalities, such as CT. Encouragingly, the studies included in this review all utilised cognitive tests with satisfactory to excellent psychometric properties [69,70,71,72,73,74,75,76,77,78,79,80]; unfortunately, the specific tests selected in the included studies were different. This may be improved in future studies by using standardised neuropsychological testing batteries, rather than relying on a more boutique selection of assessments. Several such neuropsychological batteries, such as the Cambridge Neuropsychological Test Automated Battery (CANTAB), have been previously validated in paediatric populations, are non-linguistic and offer both in-person and online delivery options [81,82,83,84], making them potentially good candidates for future investigations assessing the benefits of CT interventions in this population.

While using tests with good psychometric properties is essential, it is also important to ensure that studies are designed to optimise the performance of these assessments. This can be done by including multiple testing baselines to stabilise test outcome scores and ensuring that included tests are practice-insensitive [85,86]. Including a control group to provide insight into any placebo or practice effects that may occur is also essential. Concerningly, two of the included studies in this review did not include a control group [48,49], but rather simply compared cognition between baseline and post-intervention follow-up in the intervention group [48,49]. This may lead to false positives in interpretation of the results. In line with this, Butler et al. [46] found significant improvements in cognition post-intervention in both the experimental and control groups, with no difference between the two groups, suggesting that positive impacts may not be due to the intervention itself. Rather, these might be due to practice effects due to repeated cognitive testing.

In all the studies included in the current review, participants had a prior exposure to the cognitive assessment battery prior to evaluation of the benefits of the CT intervention. It is therefore reasonable to assume that practice effects may have, at least to some degree, influenced performance on post-intervention assessments. Such practice effects are of significant concern in cognitive testing, with research finding they are most significant in high-frequency testing (<3 months) and are most pronounced at initial exposures [87,88,89]. We recommend the inclusion of multiple baseline testing points to ensure practice gains have plateaued before attempting to measure any change related to the CT. Research has also found that the magnitude of practice effects vary depending on a range of test factors [87,88,90]. For example, test type has been shown to have a significant impact on the presence of practice effects, with tests that are stable over time, or which are practice-insensitive, being the most effective at reducing these effects [85,86]. Additionally, tests that are not novel in their design, meaning that they test skills regularly used in everyday life, have also been shown to reduce these effects, as a result of the decreased initial learning curve [85,86]. The use of alternate test forms and appropriate statistical modelling, such as Reliable Change Indices, to account for improvements over time wherever possible, may also help minimise practice effects [85,86].

In addition to practice effects, placebo effects may also contribute to the similar effects observed between the experimental and control groups, with prior studies demonstrating a positive effect for cognitive function from simply participating in research [91]. This has led some researchers to conclude that improvements seen due to CT are actually placebo effects due to positive participant expectations [91]. A subsequent study, however, looking specifically at the effects of participant expectations failed to support that training-related improvements are solely due to placebo effects [86]. Similarly, a meta-analysis of the literature reported that more intense placebos, with greater believability, did not consistently result in more beneficial effects than less intense placebos [92]. One solution that has been proposed to mitigate this issue is the inclusion of an active control group, who participate in a sterile intervention, rather than a passive control group, who only undertake cognitive testing sessions, without further engagement with the study protocol or contact with researchers [93]. Nevertheless, the benefits of this control group design are not entirely conclusive within the CT literature, with Green et al. [94] acknowledging their contentious nature and discussing that there have been significant issues with the definition of an active control in CT research. Similarly, a meta-analysis by Rodas et al. [95] concluded that an active control group did not provide significant improvement compared to studies with a passive control group. Given this uncertainty regarding current best practice, we recommend the inclusion of both active and passive control groups in future CT studies in this population—as well as within the CT literature more broadly—to assess for the potential impact of placebo effects.

Overall, there are several limitations in the current literature that became clear through the completion of this systematic review. First, it is difficult to conclude whether the benefits of using CT in childhood cancer survivors persist long-term. The studies included in this review used a mixture of three different testing time points: immediately post-intervention, at three-month follow-up and at 6-month follow-up, with no study assessing longer than 6-months following intervention completion. Two of the included studies within this review reported that improvements in working memory function persisted at the 6-month follow-up assessment [44,49], although one was an uncontrolled within-group assessment [49]. In the controlled study, this result was specific to the spatial span backward subtest of working memory, while fading in other subtests. Another three studies reported a similar sustained improvement, observed by moderate to large effects, at three months [45,47,48], reflected in 95% confidence intervals that exclude 0, again with one being uncontrolled [48]. This was not consistent across subtests though, with many now indicating an improvement. This discrepancy is in line with the wider CT literature, where the maintenance of cognitive improvement resulting from CT has been shown to be possible [96,97,98], but not universal [60]. Given this uncertainty, longitudinal study designs that include long-term follow-up out to a year or more post-intervention are critical.

In addition, it is difficult to comment on near vs. far transfer effects associated with CT in childhood cancer survivors based solely on the studies included in the current review. Within the cognitive literature, near transfer refers to when the cognitive domains trained in CT are also measurable on closely related tasks, while the term far transfer is used to indicate the transfer of skills to unrelated or very weakly related cognitive domains or on clinically meaningful outcomes, such as symptoms experienced or overall function [99,100]. Many prior studies in the CT field more broadly have reported benefits in near transfer, which is in line with the cognitive benefits reported in the studies included within this review, where all but one of the improvements detected were in domains that were directly trained. Butler et al. [46] was the only study that reported benefits in a domain that was not explicitly trained (working memory), but this would still be considered near transfer, as the system trained focused, sustained, selective, alternating and divided attention [101], which depend on working memory. This study’s finding was also not significant, with a negligible effect size, when compared to the control group. In contrast, many studies within the wider literature have been unable to demonstrate far transfer (for commentary, see [102]). Far transfer measures of academic achievement were measured in three of the studies included in this review [44,46,49], with one reporting no significant improvement [44], one reporting improvement relative to controls (despite there being no significant improvement on cognitive test performance relative to controls) [46], and the final study reporting an improvement in applied math, but with no control group available to confirm this finding [49]. The combination of these inconsistent results and significant methodology limitations highlights an important gap to be addressed in future work.

Peckham et al. [103] offer three broad ideas for how this might be improved, including: (1) better personalisation, responsive to the needs of the individual, (2) enhanced delivery of interventions “at the right time and place” to enact changes in the desired outcomes, and (3) a focus on improving the multiculturalism of CT paradigms [103]. These suggestions are further complemented by recent work focusing on how to better define transfer effects [104] and improve CT methodology overall [100]. Future research should take these into account when designing studies to assess potential far transfer benefits, as this is critical for translation of CT paradigms into clinically meaningful, day-to-day benefits for childhood cancer survivors.

When aiming to improve CT design more generally, one factor that may be critical is the dosage of the intervention. There are relatively few studies that discuss the specific impact that dose has on the benefits of CT, with, to the best of our knowledge, only one study directly investigating this relationship [105]. In this study, Liu and colleagues observed a dose-dependent effect of CT on mild cognitive decline in middle-aged to older adults. They concluded that 6 days per week of training for 25–30 min per day was optimal for their cohort aged between 40 and 60 years. While it is unclear whether the same holds true in a paediatric population, these results nevertheless indicate that differences in CT dosage can impact treatment efficacy and provide an indication that there is a ‘sweet spot’ for both frequency and length of training sessions, where too high or low dosage in either or both frequency and length will negatively impact results.

In the current review, the dosage of each intervention reported in the included studies varied substantially, ranging from 5–8 weeks to 40 weeks, with weekly training sessions ranging from 50 min per week to four 2 h sessions per week (8 h in total). The total training hours ranged from 10 to 40 h between studies. This inconsistency in training times between studies makes comparisons of treatment efficacy between studies challenging. A study investigating training time, focused on determining the optimal CT “dosage” for this population, is recommended for future work in this area, not only to improve CT research comparability, but also to manage patient burden and expectations, considerations that are especially important in a paediatric population.

Investigation into the impact “dose” has on CT efficacy is particularly relevant given that the training regimen and associated participant burden can impact compliance, and hence the effective “dose” of the CT ultimately received by participants. In line with this, prior literature in elderly adults has reported that increased engagement with a CT intervention results in increased training-related gains, particularly when compliance rates exceed 80% [106,107]. Compliance within the studies included in this review varied significantly from 44% [48] to 90.47% [49]. The three studies with the lowest compliance (Hardy et al. [48] at 44%, Benzing et al. [47] at 47.8% and Butler et al. [46] at 60%) were delivered in different settings, with one delivered in-clinic [46] and two at home [47,48]. Notably, the study with the highest compliance (90.47%) was also delivered at home [49], suggesting that the setting of training may not noticeably impact compliance. In fact, the only study feature that seemed to potentially correlate with improved compliance was more frequent engagement with CT. In line with this, Hardy et al. [45], Conklin et al. [44], Carlson-Green et al. [49] all had compliance results greater than 80%, with a prescribed dosage of at least three training sessions per week. Conversely, two of the studies with the lowest compliance [46,48] called for only one weekly session. This trend is contradicted by Benzing et al. [47] who reported one of the lowest compliances, with a substantially higher prescribed dosage. It is plausible, although the size of this data set means no conclusions can be made, that increased regular engagement with the CT paradigm encouraged participants to incorporate this into their regular routines, although Benzing et al. [47] contradicts this. No clear reason for the significant variation in compliance could be found.

It is also important to note that five of the included studies used external motivation for their participants in the form of gift cards, granting these when the participants reached certain dosage milestones [44,45,47,48,49]. This form of participant remuneration has the benefit of increasing research participant compliance [108], which may, in turn, result in greater gains from the CT paradigm. Unfortunately, however, this also introduces uncertainty surrounding authentic engagement and expected compliance with use of these interventions in a clinical, real-life setting. It is important when testing these interventions that the long-term usage goals are considered, where incorporation of the intervention into regular lifestyle routines is optimal. Rather than including external motivation to achieve the desired dosage, it is recommended that CT interventions are designed with in-built motivational features, such as gamification [109], competition and collaboration with friends and family [110], as these will encourage users to reach the desired dosage in a setting that mimics its eventual real-world usage. Notably, gamification of CT, such as the gamified digital format utilised in some capacity by all of the interventions included in the current review, has been reported to increase engagement and enjoyment, possibly improving study retention and compliance [111].

**Figure 4:**
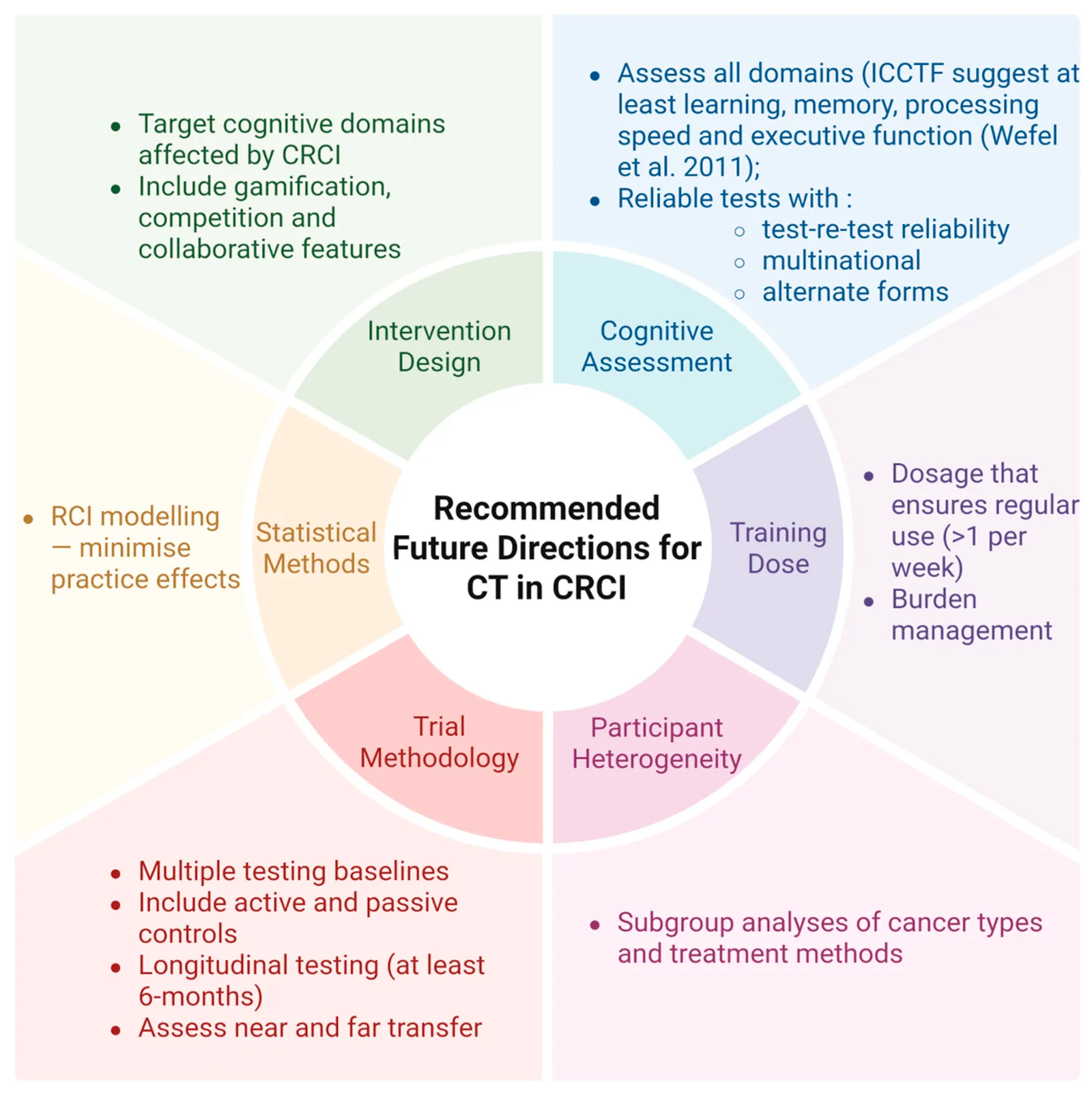
Recommended Future Directions for CT in CRCI. Created in BioRender. Collins-Praino, L. (2026) https://BioRender.com/dg1admv.

Based on our consideration of the current state of the literature, we have formulated best-practice guidelines to improve future research in this area (see Figure 4).

## 5. Conclusions

CT has been proposed as a beneficial therapeutic strategy for CRCI in the paediatric population, but, to date, only a limited number of studies have investigated its benefits. Encouragingly, five of the studies included in the current review saw a moderate to large improvement in at least one of their cognitive measures in the domain of working memory/executive function, providing preliminary evidence of the benefits of CT in this population. However, precision in these effects, primarily due to small sample sizes, heterogeneity and risk of bias, are all serious to very serious concerns, reflected in the GRADE assessment, substantially limiting the certainty of these outcomes. Unfortunately, the other two domains assessed (attention and delayed recall (memory)) did not show sufficient evidence for cognitive improvement, likely due to the limited number of studies that actually assessed these. There was also substantial variability between studies, making direct comparisons difficult, and substantial gaps in the field remain. To best optimise and standardise CT for investigation in the paediatric CRCI population in future studies, we presented a number of recommendations, including: (1) targeted training across the multiple cognitive domains impacted by CRCI; (2) inclusion of comprehensive cognitive tests standardised for use in children, with strong psychometric properties and alternate forms available; (3) optimised testing and interventions protocols, including baseline testing, suitable intervals between follow-ups and inclusion of appropriate control groups (including an active control); (4) longitudinal follow-up with investigation of near and far transfer effects; and (5) strong focus on increasing compliance, including optimised training paradigms and incorporation of intrinsic motivators, such as gamification. Taken together, we hope that these recommendations will assist in improving the quality of future research evaluating the benefits of CT for paediatric CRCI. This may, in turn, lead to the implementation of a clinically meaningful therapeutic tool to address the cognitive concerns experienced by a significant number of childhood cancer survivors.

## Figures and Tables

**Table 1:** Advanced search terms.

Category	Search Terms
Cognitive impairment/remediation	cognitive impairment* OR brain train* OR cognition disorder* OR memory disorder* OR learning disability* OR cognitive train* OR cognitive rehab* OR cognitive remediation OR cognitive dysfunction OR neurocognitive disorder*
AND	
Cancer	neoplasms OR “cancer survivors” OR cancer OR oncology OR leukemia OR leukaemia
AND	
Paediatric	child OR adolescent OR students OR infant OR pediatric OR paediatric OR teen* OR youth OR child*

**Table 4:** Attention result breakdown.

Study	Outcome	Post *d/β* (95% CI)	Post *p*	LTFO ^2^ *d/β* (95% CI)	LTFO *p*
Butler et al. [46] ^1^	Focused attention (composite)	0.02 (−0.19, 0.23)	0.879	—	—
	Vigilance (composite)	0.10 (−0.15, 0.34)	0.451	—	—
Conklin et al. [44,54], Fournier-Goodnight et al. [55], Mendoza et al. [56] ^1^	CPT-II ^3^ Omissions	**−0.61 (−1.12, −0.10)**	**0.015**	−0.25 (−0.75, 0.25)	0.055
	CPT-II ^3^ Hit Reaction Time	−0.43 (−0.93, 0.07)	**0.015**	−0.24 (−0.74, 0.26)	0.306

^1^ Follow-up at 6-months post-intervention, ^2^ LTFO—Long-Term Follow-up; ^3^ CPT—II—Conners’ Continuous Performance Test–Second Edition. Bold values indicate statistically significant results.

**Table 5:** Delayed recall (memory) result breakdown.

Study	Outcome	Post *d/β* (95% CI)	Post *p*	LTFO ^2^ *d/β* (95% CI)	LTFO ^2^ *p*
Butler et al. [46] ^1^	Memory Recall (composite)	−0.11 (−0.36, 0.14)	0.371	—	—

^1^ Follow-up at 6-months post-intervention, ^2^ LTFO—Long-Term Follow-up.

## Data Availability

This study is a systematic review of previously published research. All data supporting the findings are publicly accessible within the included studies, which are cited in the reference list. Further details are available from the corresponding author upon request.

## Supplementary Materials

The following supporting information can be downloaded at https://www.mdpi.com/article/10.3390/children13101397/s1. Supplementary Material S1: Full database search; Supplementary Material S2: Data extraction template; Supplementary Material S3: Study summaries, Supplementary Material S4: Effect estimate plots [44,45,46,47,48,49]; Supplementary Material S5: GRADE summary of findings; Supplementary Material S6: Full rationale for risk of bias judgements.

## Abbreviations

The following abbreviations are used in this manuscript:

ALL	Acute Lymphoblastic Leukaemia
AWMA	Automated Working Memory Assessment
CANTAB	Cambridge Neuropsychological Test Automated Battery
CNS	Central Nervous System
CPT	Conners’ Continuous Performance Test
CRCI	Cancer-Related Cognitive Impairment
CT	Cognitive Training
DKEFS CWIT	Delis–Kaplan Executive Function System Color Word Interference Test
GRADE	Grading of Recommendations Assessment, Development and Evaluation
ICCTF	International Cognition and Cancer Task Force
LTFO	Long-Term Follow-Up
PNET	Primitive Neuroectodermal Tumour
PRISMA	Preferred Reporting Items for Systematic reviews and Meta-Analyses
RAVLT	Rey Auditory Verbal Learning Test
RCT	Randomised controlled trial
ROB 2	Cochrane Risk Of Bias 2
SWiM	Synthesis without Meta-Analysis
ROBINS-I	Risk Of Bias In Non-randomised Studies-of Interventions
WISC	Wechsler Intelligence Scale for Children
WMTB-C	Working Memory Test Battery-Children
WRAML	Wide Range Assessment of Memory and Learning
