# Supplementary material for: How the Design of Technology-Mediated Cognitive Training Paradigms Impacts Efficacy in Childhood Cancer Survivors: A Systematic Review
Source: Children (Basel). 2026 Oct 14;13(10):1397. doi: 10.3390/children13101397 (PMC13703704; doi:10.3390/children13101397)
Supplement: Supplementary file 1 [file children-13-01397-s001.zip › children-4559966-supplementary.pdf]

Supplementary Material S1. Full database search.

	Cognitive training		Cancer		Paediatric
**Pubmed**	(“cognitive impairment*” OR “brain train*” OR “cognition disorder*” OR “memory disorder*” OR “learning disabilit*” OR “cognitive train*” OR “cognitive rehab*” OR “cognitive remediation” OR “cognitive dysfunction” OR “neurocognitive disorder*”) **[Title/Abstract]**<br>OR<br>(“chemotherapy related cognitive impairment/prevention and control” OR “Chemotherapy-Related cognitive dysfunction/rehabilitation” OR “chemotherapy-related cognitive impairment/therapy” OR “cognitive dysfunction/prevention and control” OR “cognitive dysfunction/rehabilitation” OR “cognitive dysfunction/therapy” OR “cognition disorders/prevention and control” OR “cognition disorders/rehabilitation” OR “cognition disorders/therapy” OR “neurocognitive disorders/prevention and control” OR “neurocognitive disorder-s/rehabilitation” OR “neurocognitive disorders/therapy” OR “learning disabilities/prevention and control” OR “learning disabilities/rehabilitation” OR “learning disabilities/therapy” OR “memory disorders/prevention and control” OR “memory disorders/rehabilitation” OR “memory disorders/therapy”) **[MeSH Terms]**	AND	(neoplasms OR “cancer survivors” OR cancer OR oncology OR leukemia OR leukaemia) **[Title/Abstract]**<br>OR<br>(Neoplasms/-complications* OR Neoplasms/psychology OR Neoplasms/rehabilitation OR “cancer survivors”) **[MeSH Terms]**	AND	(child OR adolescent OR students OR infant OR pediatric OR paediatric OR teen* OR youth OR child*) **[Title/Abstract]**<br>OR<br>(child OR adolescent OR students OR infant) **[MeSH Terms]**
**PsycInfo**	cognitive impairment/ OR exp Cognitive Processes/ OR exp cognitive ability/ OR learning disabilities/ OR memory disorders/ OR exp cognitive rehabilitation/ OR exp brain training/learning	AND	exp Neoplasms/ OR exp Oncology/ OR exp Leukemias/<br>OR<br>(“neoplasm*” OR “cancer” OR “oncology”	AND	exp Students/ OR exp Pediatrics/<br>OR<br>(adolescent OR students OR infant OR pediatric OR paediatric OR

	disabilities/ OR Neurocognitive Disorders/<br>OR<br>("brain training" OR "memory disorder*" OR "cognitive impairment" OR "cogn* remediation" OR "cogn* rehab*" OR "cogn* train*" OR "cognition disorder*" OR "learning disabilit*" OR "cognitive dysfunction" OR "neurocognitive disorder").**mp**		OR "leuk?emia" OR "cancer survivor*").**mp**		adolescent OR teen* OR youth OR child*).**mp**
**Scopus**	**TITLE-ABS-KEY** ("cogn* remediation" OR "cogn* rehab*" OR "cogn* train*" OR "cogn* disorder*" OR "learning disabilit*" OR "memory disorder*" OR "brain train*" OR "cogn* impairment" OR "cogn* dysfunction" OR "neurocognitive disorder*")	AND	**TITLE-ABS-KEY** (neoplasm* OR "cancer survivor*" OR cancer OR oncology OR leuk*emia)	AND	**TITLE-ABS-KEY** (child* OR adolescent OR students OR infant OR pediatric OR paediatric OR teen* OR youth)
**CINAHL**	"neurocognitive disorder" OR "cogn*dysfunction" OR "learning disabilit*" OR "cogn* disorder*" OR "cogn* train*" OR "cogn* rehab*" OR "cogn* remediation" OR "cognitive impairment" OR "memory disorder*" OR "brain training"<br>OR<br>**MH** ("Memory Disorders/PC/RH/TH" OR "Learning Disorders/TH/RH/PC" OR "Memory Disorder-s/TH/RH/PC" OR "Cognition Disorders/RH/PC/TH" OR "Chemotherapy-Related Cognitive Impairment/RH/PC/TH" OR "Mild Cognitive Impairment/RH/PC/TH" OR "Rehabilitation, Cognitive" OR "Cognitive Remediation")	AND	"leuk#emia" OR "oncology" OR "cancer" OR "cancer survivor*" OR "neo-plasm*"<br>OR<br>**MH** ("Leukemia" OR "Oncology" OR "Cancer Survivors" OR "Neoplasms/-CO/RH/PF")	AND	"adolescen*" OR "student*" OR "infant" OR "p#ediatric" OR "teen*" OR "youth" OR "child"<br>OR<br>**MH** ("Adolescence" OR "Infant" OR "Students" OR "Child")

Supplementary Material S2. Data extraction template

Identification:
- Sponsorship source
- Country
- Setting
- Comment
- **Authors contact details:**
  - Name
  - Institution
  - Email
  - Address

Methods:
- Study design
- Group
- **Additional data:**
  - Aim
  - Timepoint(s) investigated
  - Cognitive training design (basic)
  - Cognitive domains targeted
  - External motivation

Population:
- Inclusion criteria
- Exclusion criteria
- Group differences
- **Baseline characteristics:**
  - Age
  - Gender
  - Cancer diagnosis
  - Treatment received
  - Time since cessation of treatment/in remission
  - Total sample size (n =)

Interventions:
- Cognitive training
- Control/comparator
- **Intervention details:**
  - Difficulty progression
  - Visual/auditory stimuli
  - Competition
  - Any additional information needed
  - Cognitive training design (digital, pen/paper)
  - Timeline of cognitive training
  - Cognitive domains targeted

Outcomes:
- Cognitive test measures
- Compliance

Supplementary Material S3. Study summaries

Study	Study design	Sample size	Population summary (gender and age)	Cancer diagnosis & time since treatment cessation	Treatment summary	Cognitive training & dose	Timepoints tested
Butler et al [46]	Parallel group RCT[1]	Intervention:		Cancer diagnosis and timeline not reported	Not reported	Cognitive Remediation Program 20 weekly sessions (2-hours each) over 4-5 months in a clinic settings	Before intervention, immediately after intervention, 6-month follow-up (not included in publication statistical modelling)
		108 at baseline, 90 at immediate follow-up, 80 at long-term	38% female, 62% male age: 10.8 ± 3.4				
		Control:					
		53 at baseline, 52 at immediate follow-up	30.2% female, 69.8% male age: 11.1 ± 3.1				
Hardy et al. [48]	Self-controlled pilot trial	Intervention:				Captains Log At least 50 minutes per week for 12 weeks at home	Before intervention, immediately after intervention, 3-month follow-up
		9 at all stages	4 female, 5 male age: 13.3 ± 2.44	Cancer types: 3 ALL[2], 2 Ependymoma, 4 Medulloblastoma/PNET[3] Time since treatment 5.7 ± 3.2 (1-10 years)	5 CR[4], 8 chemo[5], 6 surgery		
Hardy et al. [45]	Parallel group RCT[1]	Intervention:				Cogmed RM 25 sessions (3-5 sessions per week) over 5-8 weeks at home	Before intervention, immediately after intervention, 3-month follow-up
		13 at baseline, 11 at follow-up points	5 female, 8 male age: 12.7 ± 2.77	Cancer types: 7 ALL[2], 3 Ependymoma, 2 Medulloblastoma/PNET[3], 1 Other Time since treatment: 6 ± 2.98 years	5 neurosurgery, 10 chemo[5], 5 CR[4]		
		Control:					
		7 at baselines, 6 at follow-up points	3 female, 4 male age: 10.7 ± 1.89	Cancer types: 4 ALL[2], 2 Medulloblastoma/PNET[3], 1 Other Time since treatment: 3 ± 1.77 years	3 Neurosurgery, 6 chemo[5], 4 CR[4]		
		Intervention:					

Conklin et al. [44, 54], Fournier-Goodnight et al. [55], Mendoza et al. [56]	Parallel group RCT[1]	34 at baseline, 30 at immediate and long-term follow-up	16 female, 18 male age: 12.21 ± 2.47	Cancer types: 23 ALL[2], 1 Ependymoma, 8 Medulloblastoma/PNET[3], 1 Glioma Time since treatment: 4.97 ± 3.02 years	20 chemo[5] only, 8 CR[4] & chemo[5], 3 CRT[6] & chemo[5], 3 chemo[5] & BMT[7] & TBI[8]	Cogmed 25 sessions over 5-9 weeks, each 30-45 minutes, at home	Before intervention, immediately after intervention, 6-month follow-up
		Control:					
		34 at baseline, 32 at immediate follow-up, 30 at long-term follow-up	16 female, 18 male age: 11.82 ± 2.42	Cancer types: 24 ALL[2], 7 Medulloblastoma/PNET[3], 3 Ependymoma Time since treatment: 5.04 ± 2.41 years	22 chemo[5] only, 7 CR[4] & chemo[5], 3 CRT[6] & chemo[5], 2 chemo[5] & BMT[7] & TBI[8]		
Carlson-green et al. [49]	Prospective self-controlled pilot trial	Intervention:				Cogmed 35 sessions over 8-12 weeks at home	Before intervention, immediately after intervention, 6-month follow-up
		21 at baseline, 20 at immediate follow-up, 19 at long-term follow-up	55% female, 45% male age: 8-18 years	Cancer types: 4 Ependymoma, 11 Medulloblastoma/PNET[3], 4 Germinoma, 2 Other Time since treatment: mean 5 years (1-12 years)	Surgery, chemo[5] and radiation		
Benzing et al. [47]	Parallel group RCT[1]	Intervention:				Cogmed 45 minutes per session, 3 times per week for 8 weeks	Before intervention, immediately after intervention, 3-month follow-up
		23 at baseline, 20 at immediate follow-up, 15 at long-term follow-up	11 female, 12 male age: 10.71 ± 2.48	Cancer types: 15 leukaemia and lymphomas, 4 CNS[9] tumours and neuroblastomas, 4 Other Time since treatment not reported	2 radiation, 10 chemo[5], 2 surgery & radiation, 4 surgery & chemo[5], 5 chemo[5] & radiation & surgery		
		Control:					
		24 at baseline, 22 at immediate follow-up, 17 at long-term follow-up	8 female, 16 male	Cancer types: 12 leukaemia and lymphomas, 5 CNS[9] tumours and neuroblastomas, 7 Other Time since treatment not reported	1 radiation, 10 chemo[5], 6 surgery & chemo[5], 7 chemo[5] & radiation & surgery		

[1] Randomised controlled trial (RCT)

[2] Acute lymphoblastic leukaemia (ALL)

[3] Primitive neuroectodermal tumour (PNET)

[4] Cranial radiation (CR)

[5] Chemotherapy (chemo)

[6] Conformal Radiation therapy (CRT)

[7] Bone marrow transplant (BMT)

[8] Total body irradiation (TBI)

[9] Central nervous system (CNS)

Supplementary Material S4. Effect estimate plots [44–49]

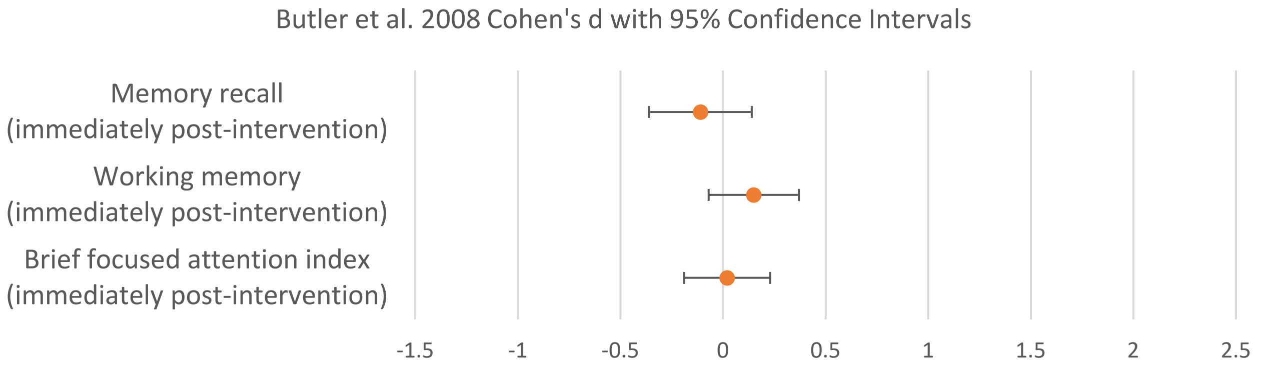

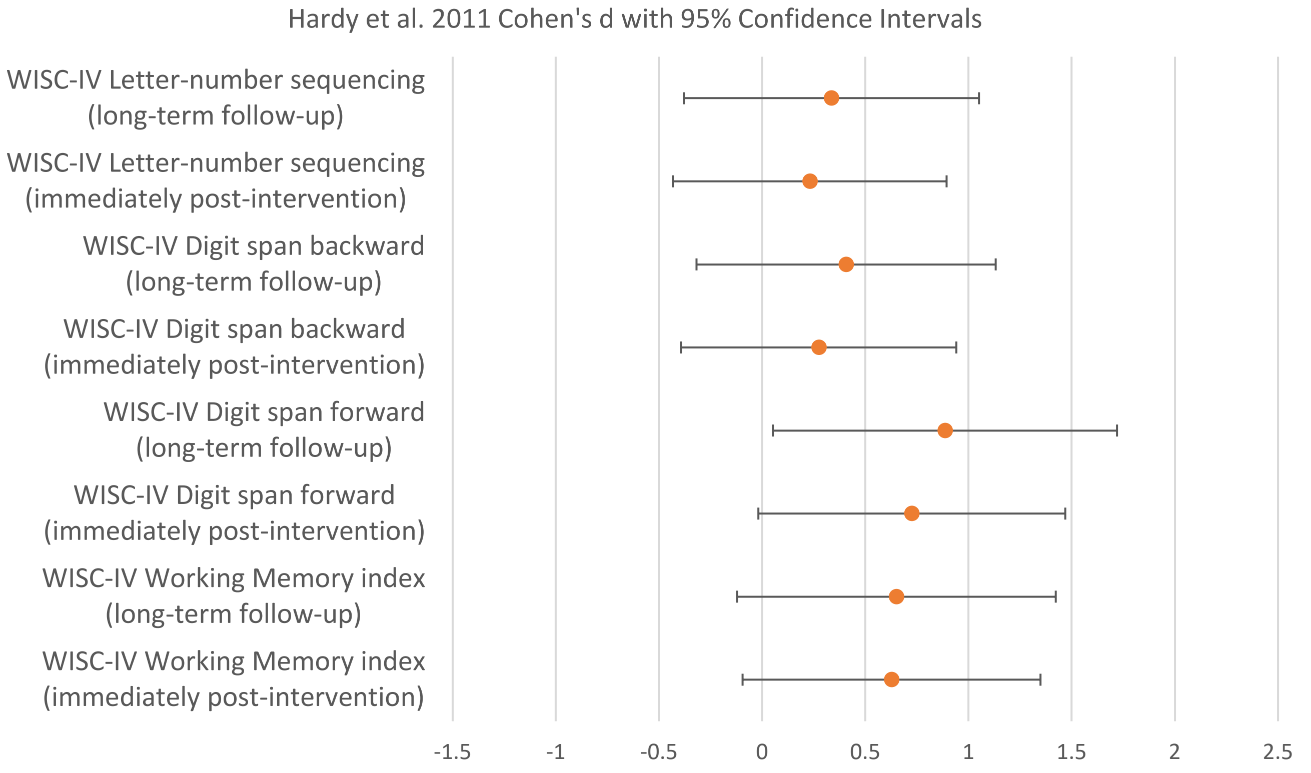

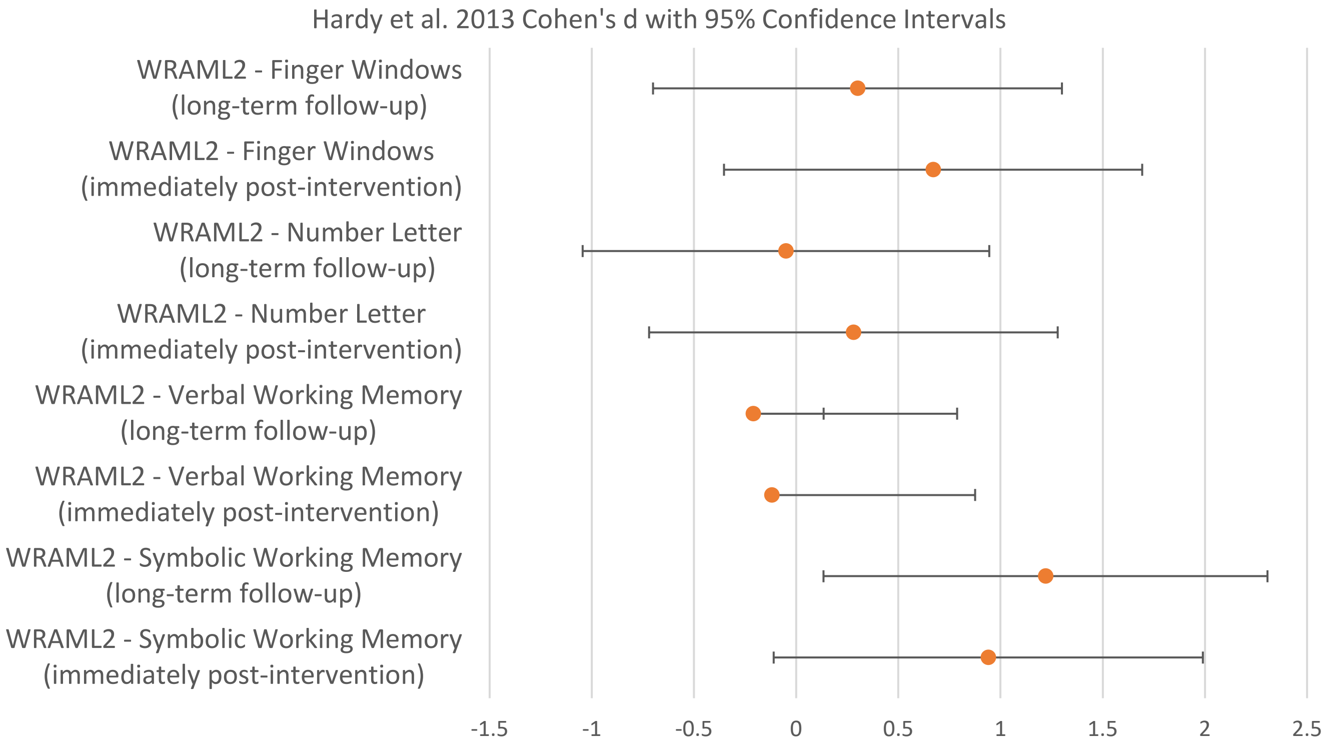
Hardy et al. 2013 Cohen's d with 95% Confidence Intervals
WRAML2 - Finger Windows (long-term follow-up)
WRAML2 - Finger Windows (immediately post-intervention)
WRAML2 - Number Letter (long-term follow-up)
WRAML2 - Number Letter (immediately post-intervention)
WRAML2 - Verbal Working Memory (long-term follow-up)
WRAML2 - Verbal Working Memory (immediately post-intervention)
WRAML2 - Symbolic Working Memory (long-term follow-up)
WRAML2 - Symbolic Working Memory (immediately post-intervention)
-1.5
-1
-0.5
0
0.5
1
1.5
2
2.5

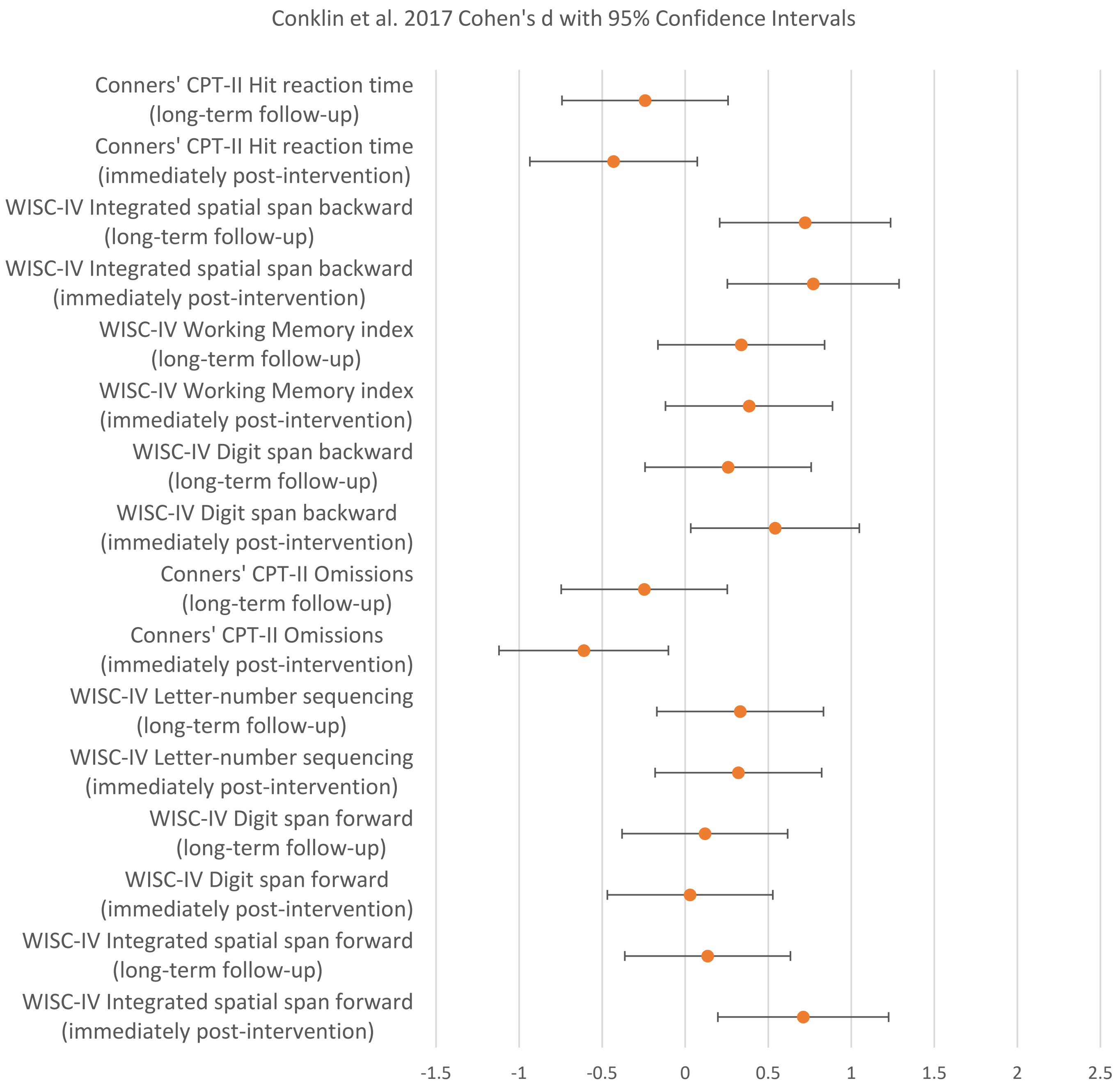
Conklin et al. 2017 Cohen's d with 95% Confidence Intervals
Conners' CPT-II Hit reaction time (long-term follow-up)
Conners' CPT-II Hit reaction time (immediately post-intervention)
WISC-IV Integrated spatial span backward (long-term follow-up)
WISC-IV Integrated spatial span backward (immediately post-intervention)
WISC-IV Working Memory index (long-term follow-up)
WISC-IV Working Memory index (immediately post-intervention)
WISC-IV Digit span backward (long-term follow-up)
WISC-IV Digit span backward (immediately post-intervention)
Conners' CPT-II Omissions (long-term follow-up)
Conners' CPT-II Omissions (immediately post-intervention)
WISC-IV Letter-number sequencing (long-term follow-up)
WISC-IV Letter-number sequencing (immediately post-intervention)
WISC-IV Digit span forward (long-term follow-up)
WISC-IV Digit span forward (immediately post-intervention)
WISC-IV Integrated spatial span forward (long-term follow-up)
WISC-IV Integrated spatial span forward (immediately post-intervention)
-1.5
-1
-0.5
0
0.5
1
1.5
2
2.5

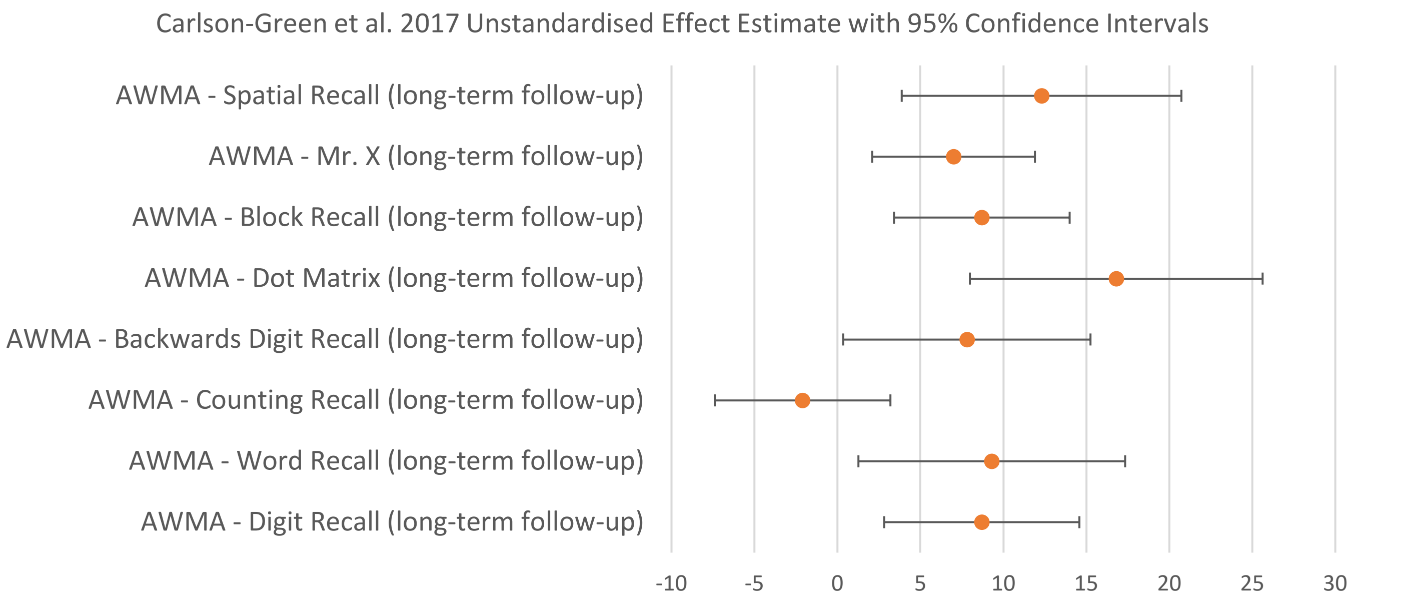
Carlson-Green et al. 2017 Unstandardised Effect Estimate with 95% Confidence Intervals
AWMA - Spatial Recall (long-term follow-up)
AWMA - Mr. X (long-term follow-up)
AWMA - Block Recall (long-term follow-up)
AWMA - Dot Matrix (long-term follow-up)
AWMA - Backwards Digit Recall (long-term follow-up)
AWMA - Counting Recall (long-term follow-up)
AWMA - Word Recall (long-term follow-up)
AWMA - Digit Recall (long-term follow-up)
-10
-5
0
5
10
15
20
25
30

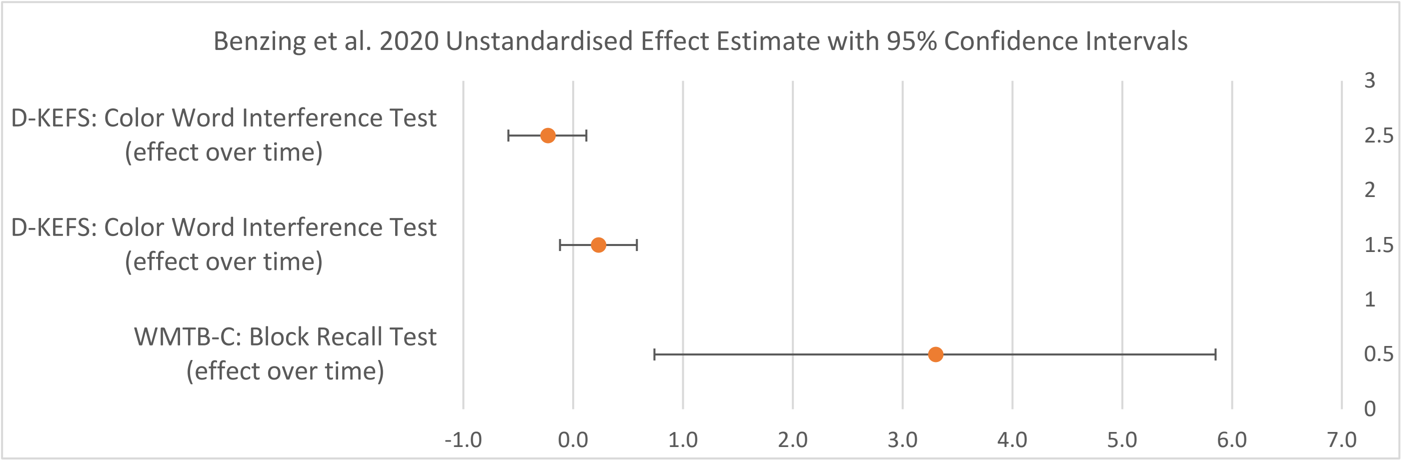
Benzing et al. 2020 Unstandardised Effect Estimate with 95% Confidence Intervals
D-KEFS: Color Word Interference Test (effect over time)
D-KEFS: Color Word Interference Test (effect over time)
WMTB-C: Block Recall Test (effect over time)
-1.0
0.0
1.0
2.0
3.0
4.0
5.0
6.0
7.0
3
2.5
2
1.5
1
0.5
0

Supplementary Material S5. GRADE Summary of Findings

Certainty assessment							No of patients		Effect		Certainty	Importance
No of studies	Study design	Risk of Bias	Inconsistency	Indirectness	Imprecision	Other	Cognitive training	control	Relative (95% CI)	Absolute (95% CI)		
Working memory and executive function – immediate follow-up												
3	randomized trials	serious[1]	not serious[2]	not serious[3]	serious[4]	none	131	91	not applicable	not estimable	⊕⊕◯◯ low	CRITICAL
1	non-randomised studies	serious[5]	not estimable[6]	not serious[3]	very serious[7]	none	9	0	not applicable	not estimable	⊕◯◯◯ very low	CRITICAL
Working memory and executive function – long-term follow-up												
3	randomized trials	serious[8]	not serious[2]	not serious[3]	serious[9]	none	56	53	not applicable	not estimable	⊕⊕◯◯ low	CRITICAL
2	non-randomised studies	serious[10]	not serious[2]	not serious[3]	very serious[11]	none	28	0	not applicable	not estimable	⊕◯◯◯ very low	CRITICAL
Attention – immediate follow-up												
2	randomized trials	serious[12]	not serious[2]	not serious[3]	serious[13]	none	120	85	not applicable	not estimable	⊕⊕◯◯ low	CRITICAL
Attention – long-term follow-up												
1	randomized trials	serious[14]	not estimable[6]	not serious[3]	very serious[15]	none	30	30	not applicable	not estimable	⊕◯◯◯ very low	CRITICAL
Delayed recall (memory) – immediate follow-up												
1	randomized trials	serious[16]	not estimable[6]	not serious[3]	serious[17]	none	90	53	not applicable	d = -0.11 (-0.36, 0.14)	⊕⊕◯◯ low	CRITICAL

[1] Downgraded one level for risk of bias as all studies were judged to have some concerns. Butler et al. [46] had some concerns due to randomisation, deviations from intended interventions, missing outcome data, and selection of the reported result. Hardy et al [45] had some concerns due to missing outcome data. Conklin et al. [44] had some concerns due to missing outcome data and selection of the reported result.

[2] The direction and magnitude of the effect does not substantially differ across studies

[3] The study participants, intervention and comparators all directly address the clinical question.

[4] Imprecision was rated as serious as the total sample size across studies (intervention n = 131; control n = 91) is below the suggested threshold of 400 participants posed by Murad et al. [43], and confidence intervals were wide, including a range effects, from potential benefit to potential harm.

[5] Downgraded one level for risk of bias because the study was judged to have a serious risk of bias in ROBINS-I due to serious bias due to confounding and deviations from intended interventions, and moderate bias in measurement of outcomes and selection of the reported result.

[6] As there is only one study that assessed this domain, it is not possible to assess inconsistency.
[7] Imprecision was rated as very serious as the total sample size across studies (intervention n = 9) is below the suggested threshold of 400 participants posed by Murad et al. [43], and confidence intervals were wide, including a range effects, from potential benefit to potential harm.
[8] Downgraded one level for risk of bias as the three studies were all judged to have some concerns. Hardy et al [45] had some concerns due to missing outcome data. Conklin et al. [44] had some concerns due to missing outcome data and selection of the reported result. Benzing et al. [47] had some concerns due to missing outcome data.
[9] Imprecision was rated as serious as the total sample size across studies (intervention n = 56; control n = 53) is below the suggested threshold of 400 participants posed by Murad et al. [43], and confidence intervals were wide, including a range effects, from potential benefit to potential harm.
[10] Downgraded one level for risk of bias as both studies were judged to have a serious risk of bias in ROBINS-I. Hardy et al [48] had a serious risk of bias due to serious confounding and deviations from intended interventions, and moderate bias in measurement of outcomes and selection of the reported result. Carlson-Green et al. [49] had a serious risk of bias due to confounding.
[11] Imprecision was rated as very serious as the total sample size across studies (intervention n = 28) is below the suggested threshold of 400 participants posed by Murad et al. [43], and confidence intervals were wide, including a range effects, from potential benefit to potential harm.
[12] Downgraded one level for risk of bias as both studies were assessed to have some concerns. Butler et al. [46] had some concerns due to randomisation, deviations from intended interventions, missing outcome data, and selection of the reported result. Conklin et al. [44] had some concerns due to missing outcome data and selection of the reported result.
[13] Imprecision was rated as serious as the total sample size across studies (intervention n = 120; control n = 85) is below the suggested threshold of 400 participants posed by Murad et al. [43], and confidence intervals were wide, including a range effects, from potential benefit to potential harm.
[14] Downgraded one level for risk of bias as the study was assessed to have some concerns due to missing outcome data and selection of the reported result.
[15] Imprecision was rated as very serious as the total sample size across studies (intervention n = 30; control n = 30) is below the suggested threshold of 400 participants posed by Murad et al. [43], and confidence intervals were wide, including a range effects, from potential benefit to potential harm.
[16] Downgraded one level for risk of bias as the study was assessed to have some concerns due to randomisation, deviations from intended interventions, missing outcome data, and selection of the reported result.
[17] Imprecision was rated as serious as the total sample size across studies (intervention n = 90; control n = 53) is below the suggested threshold of 400 participants posed by Murad et al. [43], and confidence intervals were wide, including a range effects, from potential benefit to potential harm

Supplementary Material S6. Full rationale for risk of bias judgements

RoB 2.0

The RCT of Butler et al. [46] demonstrated some concerns with risk of bias for working memory at post-intervention, primarily due to deviations from intended interventions, missing outcome data, and selection of the reported result. Participants were randomly assigned by a central data manager in a 2:1 ratio, although methods for sequence generation and allocation concealment were not reported. Only 60% completed the full 20-session intervention, although 80% completed at least three quarters of the intervention. At post-intervention, completion was lower in the cognitive remediation group than the wait-list group (83% vs 98%, p=.04), with working memory data available for 90/107 and 52/52 participants, respectively. Repeated-measures models accounted for incomplete data, but differential missingness could not be excluded as a source of bias. Measurement of outcomes was assessed at low risk due to the use of standardised, performance-based neuropsychological measures with objective scoring. Some concerns were also identified for selection of the reported result because, although five cognitive indices were reportedly prespecified in the protocol, no protocol or prospective analysis plan was available for verification.

The RCT of Hardy et al. [45] demonstrated some concerns with risk of bias for symbolic working memory, verbal working memory, number-letter, and finger windows at post-intervention and 3-month follow-up, primarily due to missing outcome data. Three noncompliant participants did not return for follow-up and were excluded from the final analyses, while primary endpoint data were missing for one participant due to examiner error, with no additional analysis reported to account for missing data. The randomisation process, deviations from intended interventions, measurement of outcomes, and selection of the reported result were assessed at low risk of bias. Participants were randomised using blocked randomisation stratified by diagnosis, outcome assessors were blinded to allocation, and analyses followed a prespecified plan.

The RCT of Conklin et al. [44, 54-56] demonstrated some concerns with risk of bias for spatial span forward, digit span forward, letter-number sequencing, digit span backward, working memory index, spatial span backward, omissions, and hit reaction time at post-intervention and 6-month follow-up, due to missing outcome data and selection of the reported result. The handling of missing or partial outcome data was unclear, and partial data may have been excluded from analysis. No prespecified statistical analysis plan was available, although the primary outcomes and analysis methods were specified in the report. The randomisation process, deviations from intended interventions, and measurement of outcomes were assessed at low risk of bias, with computer-generated stratified randomisation and blinded cognitive outcome assessors.

The RCT of Benzing et al. [47] demonstrated some concerns with risk of bias for block recall, inhibition, and shifting at 6-month follow-up due to missing outcome data. Although missingness was similar across intervention groups and linear mixed models and multiple imputation were used, some reasons for noncompletion could potentially be related to outcomes and bias due to data not being missing at random could not be excluded. The randomisation process, deviations from intended interventions, measurement of outcomes, and selection of the reported result were assessed at low risk of bias. Primary outcomes and analyses were prespecified in the clinical trial registry and analyses followed an intention-to-treat approach.

ROBINS-I

The NRSI of Hardy et al. [48] demonstrated serious overall risk of bias for working memory at post-intervention and 3-month follow-up, primarily due to confounding and deviations from intended interventions. The within-participant pre-post design had no concurrent control group, meaning observed changes could reflect maturation, practice effects, regression to the mean, or other time-varying factors. Intervention exposure also varied substantially (9–53 sessions; 3.7–20.8 hours). Moderate risk of bias was also identified for measurement of outcomes and selection of the reported result due to repeated WISC-IV assessment, lack of blinding, multiple outcomes and time-related analyses, and the absence of a prospective statistical analysis plan.

The NRSI of Carlson-Green et al. [49] demonstrated serious overall risk of bias for the AWMA subtests at 6-month follow-up, primarily due to confounding. The self-controlled design had no concurrent control group, meaning observed changes could reflect maturation, practice effects, regression to the mean, or other time-varying factors rather than the intervention. The remaining domains were assessed at low risk of bias, with most participants completing the intended intervention (19/20 completed all 35 sessions) and outcomes assessed using standardised, objectively scored cognitive tests.
